# PTEN Deletion, mTORC1 Signatures and CAD Expression in Primary Prostate Cancer: Protein-Level Data and the Role of ERG Fusion

**DOI:** 10.3390/cells15211997

**Published:** 2026-10-31

**Authors:** Itika Arora, Suvasmita Rath, Qazi Mohammad Sajid Jamal, Ahmed Aljohani, Abid Hamid, Ahmed Abu-Zaid, Mohammad Imran Khan, Ahmed Yaqinuddin

**Affiliations:** 1 College of Medicine, Alfaisal University, Riyadh 11533, Saudi Arabia; itiarora@alfaisal.edu (I.A.);; 2 Department of Biotechnology, Utkal University, Vani Vihar, Bhubaneswar 751004, Odisha, India; sophy.biotech@gmail.com; 3 Department of Health Informatics, College of Applied Medical Sciences, Qassim University, Buraydah 51452, Saudi Arabia; m.quazi@qu.edu.sa; 4 College of Science and Health Professions, King Saud Bin Abdulaziz University for Health Sciences, Riyadh 11481, Saudi Arabia; johaniah@ksau-hs.edu.sa; 5 King Abdullah International Medical Research Center, King Saud Bin Abdulaziz University for Health Sciences, Ministry of National Guard–Health Affairs, Riyadh 11481, Saudi Arabia; 6 Department of Biotechnology, School of Life Sciences, Central University of Kashmir, Ganderbal 191201, Jammu and Kashmir, India; abidhamid76@gmail.com; 7 Research Center, King Faisal Specialist Hospital and Research Center, Jeddah 21499, Saudi Arabia

**Keywords:** PTEN, CAD, mTORC1, ERG fusion, de novo pyrimidine synthesis, prostate cancer, TCGA-PRAD, reverse-phase protein array, confounding, meta-analysis

## Abstract

PTEN loss activates PI3K–AKT signaling, and mTORC1 stimulates pyrimidine synthesis through S6K1-mediated CAD phosphorylation. This exploratory public-data analysis tested whether PTEN deletion in primary prostate cancer is associated with CAD, mTORC1 and pyrimidine-synthesis expression, and whether these survive protein-level and confounder analyses. In 485 primary tumors from The Cancer Genome Atlas Prostate Adenocarcinoma (TCGA-PRAD; 159 PTEN-deleted), deletion was associated with higher CAD mRNA (Hedges’ g = 0.57) and higher Hallmark mTORC1 scores (g = 0.54). In 342 tumors with reverse-phase protein-array data, deletion was accompanied by lower PTEN protein and higher AKT Ser473 phosphorylation but not by a higher pre-specified mTORC1 phospho-output composite (g = −0.02), itself uncorrelated with the transcriptional score. In a pre-specified model adding purity, TP53, RB1, ERG fusion and batch (*n* = 276), the PTEN–CAD association was absent (β = −0.002; *p* = 0.96), whereas the mTORC1-score association persisted. In a post hoc decomposition, ERG fusion, which co-occurs with PTEN deletion (odds ratio 3.6), changed the coefficient most. Across eight cohorts, a small PTEN mRNA–CAD association (g = 0.23) was attenuated to 0.14 after ERG adjustment, with overlapping confidence intervals (Hartung–Knapp *p* = 0.071). Bulk-tumor pathway signatures and PTEN–CAD associations need protein-level and ERG-aware interpretation.

## 1. Introduction

Prostate cancer is one of the most commonly diagnosed cancers and a leading cause of cancer-related mortality in men worldwide [1], and treatment-resistant disease remains a major clinical problem [2]. The tumor suppressor PTEN is deleted or mutated in 20–40% of primary prostate tumors and more often in advanced disease [3,4]. PTEN dephosphorylates phosphatidylinositol-3,4,5-trisphosphate and so restrains PI3K signaling [5,6]; its loss permits AKT activation, which activates mTORC1 by inhibiting the TSC1/TSC2–Rheb axis [7,8]. mTORC1 activates ribosomal protein S6 kinase 1 (S6K1) [9], which phosphorylates the trifunctional enzyme CAD (carbamoyl-phosphate synthetase II–aspartate transcarbamoylase–dihydroorotase) at Ser1859 and so stimulates the first steps of de novo pyrimidine synthesis [10,11,12,13]. PTEN loss has been shown to increase glutamine flux into pyrimidine synthesis and to confer sensitivity to dihydroorotate dehydrogenase inhibition [14,15], and CAD phosphorylation downstream of PI3K–AKT has been described in advanced prostate cancer and in PTEN-deleted glioblastoma stem cells [16,17].

Whether PTEN status in human prostate tumors is associated with the expression of CAD and of the pyrimidine-synthesis program has not been examined systematically. Doing so with public data raises two problems that are general to transcriptomic pathway studies. First, transcriptional “activity” signatures are statistical summaries of bulk mRNA and may not reflect measured signaling. Second, PTEN loss in prostate cancer is not randomly distributed across genomic subtypes: it is enriched in tumors carrying TMPRSS2–ERG fusions, with which it cooperates in mouse models [18,19]. Any transcript associated with ERG fusion can therefore appear to be associated with PTEN loss.

The aim of this exploratory, hypothesis-generating study was to test the association between PTEN status and CAD, mTORC1 and pyrimidine-synthesis expression in primary prostate tumors, and to examine it in relation to these two problems. We hypothesized that, if PTEN loss increases CAD expression through PI3K–AKT–mTORC1 signaling, PTEN-deleted tumors would show higher CAD mRNA and higher measured mTORC1 output, independently of co-occurring genomic alterations. At the genomic level, in TCGA-PRAD, we compared PTEN-deleted with copy-number-neutral tumors, incorporated somatic mutations, checked the transcript-level findings against reverse-phase protein array (RPPA) data from the same tumors, and adjusted for clinical covariates, proliferation, tumor purity, TP53 and RB1 status, ERG fusion and sequencing batch. We attempted replication in a non-TCGA genomic cohort (MSKCC). At the transcript level, across eight cohorts in which only PTEN mRNA is available, we compared CAD mRNA between PTEN-Low and PTEN-High primary tumors with and without adjustment for ERG mRNA. Experimental validation is outside the scope of the study, and all conclusions are limited to association. Western blots for pCAD(Ser1859), total CAD, pS6K1(Thr389), total S6K1 and β-actin were performed in a prostate epithelial cell-line series and are reported as exploratory observations in Supplementary Note S1; they do not contribute to the conclusions.

## 2. Materials and Methods

### 2.1. Study Design, Data Sources and Pre-Specification

This is a secondary analysis of publicly available, de-identified data. Eight prostate cancer transcriptomic cohorts were analyzed: TCGA-PRAD [20] and seven Gene Expression Omnibus (GEO) series (GSE21034, GSE6919, GSE35988, GSE46602, GSE70770, GSE55945 and GSE32571) [21] (Table 1). For TCGA-PRAD, RSEM-normalized expression (HiSeqV2; log_2_(normalized count + 1)), GISTIC2 gene-level thresholded copy-number calls [22] and the clinical matrix were obtained from the UCSC Xena TCGA hub [23], and gene-level read counts for the genome-wide tumor-versus-normal analysis were obtained from the Genomic Data Commons [24]. RPPA data, MC3 somatic mutation calls [25] and the progression-free interval of the TCGA Pan-Cancer Clinical Data Resource [26] were obtained from the cBioPortal TCGA PanCanAtlas study, and ABSOLUTE purity [27], ERG fusion status, TP53 and RB1 status and sequencing batch from the cBioPortal TCGA 2015 study [20,28]. The MSKCC cohort of Taylor et al. [4] (copy number and Affymetrix exon-array expression) was obtained from cBioPortal as a non-TCGA genomic cohort; its patients are the same as those of GSE21034, so it is independent of TCGA-PRAD but not of the cross-cohort analysis. GEO series-matrix values were log_2_-transformed where necessary, quantile-normalized within cohort with limma [29] and collapsed to HGNC symbols. Every effect size is computed within a cohort before pooling, so no cross-platform batch correction was applied. A non-prostate dataset (GSE138417; MCF7 cells treated with mTOR inhibitors) served as a pharmacological control (Section 2.8). Data were retrieved between April and October 2026; file sources and checksums are listed in the repository manifest.

Pre-specification. The analyses in this paper fall into three groups, and each is labeled in the Results. (i) The TCGA-PRAD genomic contrasts, signature analyses, models M1–M4 and the cross-cohort PTEN mRNA meta-analyses form the original analysis. The genomic PTEN definition was adopted after a sample-matching error in an earlier version was found, and the designation of the primary-tumor-restricted meta-analysis as the principal cross-cohort analysis was made after it and the all-sample analysis had been run; both are therefore post hoc choices. (ii) The protein-level, mutation-aware, confounder, non-TCGA-cohort, survival, CAD copy-number and alternative-signature analyses were specified in a written analysis plan committed to the project repository (commit d0118c8) before any of them was run, and are reported in full whatever their result. The plan was not registered with an external registry. (iii) After the result of (ii) was known, the confounder result was decomposed covariate by covariate and within ERG strata without a prior written plan; the subsequent ERG-proxy validation and ERG-adjusted analyses were specified in a plan addendum committed to the repository before they were run (commit 9830705). Checks of ERG measurement quality by cohort and descriptive comparisons of the analyzed subsets are also post hoc. All analyses in (iii) are labeled post hoc. Corrections relative to earlier versions of this work are documented in the repository erratum.

### 2.2. Genomic PTEN Classification in TCGA-PRAD

Analyses were restricted to primary tumors (TCGA sample type 01; *n* = 497), of which 491 had a GISTIC2 PTEN call. Tumors with a call of −2 (deep deletion) or −1 (shallow deletion) were classified as PTEN-deleted (*n* = 159; 95 deep, 64 shallow), tumors with a call of 0 as copy-number-neutral (CN-neutral; *n* = 326), and the six tumors with a gain were excluded, giving 485 tumors for every genomic contrast. In a mutation-aware definition, tumors were classified as PTEN-altered if they carried a deletion or a non-silent PTEN mutation in the MC3 calls, and as unaltered if CN-neutral and PTEN wild-type; this was applied to the 484 classified tumors in the mutation-calling set. “PTEN-deleted” and “CN-neutral” refer only to the genomic classification, and “PTEN-Low” and “PTEN-High” only to the within-cohort PTEN mRNA median split (Section 2.7).

### 2.3. Signature Scoring and Covariates

Two predefined gene sets were scored in the 497 TCGA-PRAD primary tumors with gene set variation analysis (GSVA v1.50.0; method = “gsva”, kcdf = “Gaussian”, minimum set size 5) [30]: HALLMARK_MTORC1_SIGNALING [31] with RRM2 removed (the only gene it shares with the pyrimidine set; 199 genes, all present after mapping nine current symbols to the HiSeqV2 annotation), and a de novo pyrimidine-synthesis set (CAD, DHODH, UMPS, CTPS2, RRM1, RRM2, TK1, TYMS, DTYMK; CTPS1 is absent from HiSeqV2) [32] (gene lists in Table S1; single-sample GSEA [33] re-scoring in Table S2). A proliferation score (mean Z-score of MKI67, TOP2A, CCNB1, BUB1, AURKA, PLK1, CDK1, CENPF, UBE2C, BIRC5) and an androgen-receptor (AR) activity score (KLK3, KLK2, NKX3-1, TMPRSS2, FKBP5, PLPP1, NDRG1, STEAP4, ABCC4, ZBTB16) were computed as covariates. Seven further mTORC1- or SREBP-related gene sets from MSigDB c2 (names containing MTOR, RAPAMYCIN, or SREBF, at least 10 genes) [31,34] were scored in the same way as alternative signatures.

### 2.4. Protein-Level Analysis

RPPA data were the TCGA PanCanAtlas level-4 values (log_2_ scale, normalized across the pan-cancer RPPA set by replicate-based normalization) [35], as distributed by cBioPortal (file data_rppa.txt, study prad_tcga_pan_can_atlas_2018). The file contains one profile per tumor sample, so no replicate arrays had to be merged. Values were available for 342 classified primary tumors (118 PTEN-deleted, 224 CN-neutral). The pre-specified family comprised PTEN; AKT pSer473 and pThr308 and mTOR pSer2448 (upstream); and six mTORC1 outputs: p70S6K pThr389, S6 pSer235/236 and pSer240/244, and 4E-BP1 pSer65, pThr37/46 and pThr70. The pre-specified primary protein endpoint was an mTORC1 phospho-output score, the mean Z-score of the six output antibodies. Contrasts used Hedges’ g and the Wilcoxon test with Benjamini–Hochberg FDR within the family; all 193 antibodies with data were tested as a separate exploratory family. CAD is not on the RPPA panel.

### 2.5. Differential Expression and Correlation

**Figure 1:**
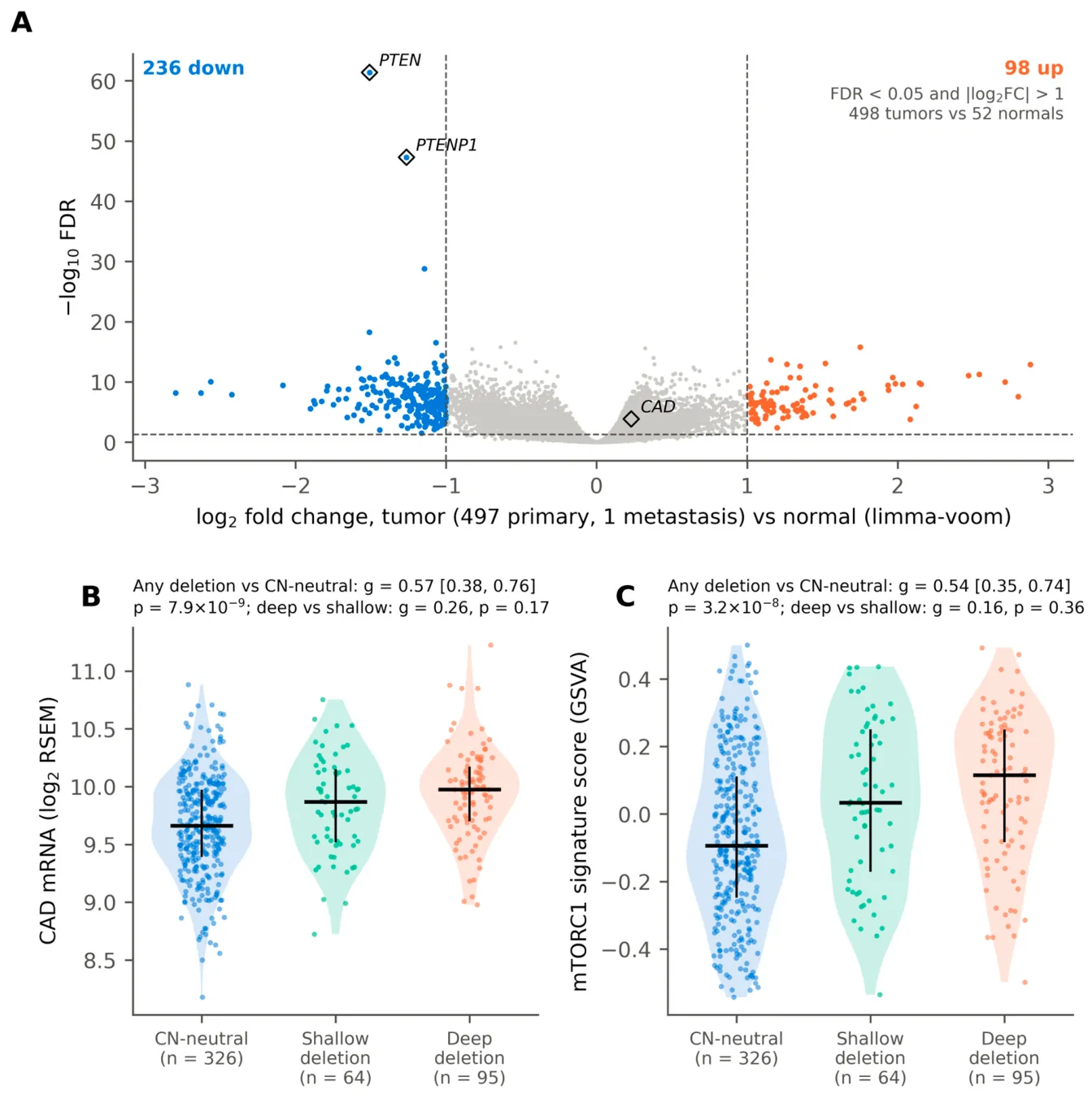
Tumor-versus-normal expression and PTEN deletion in TCGA-PRAD. (**A**) Genome-wide limma-voom differential expression, tumor (*n* = 498: 497 primary tumors and one metastasis) versus normal prostate (*n* = 52); 236 genes down and 98 up at FDR < 0.05 and |log_2_FC| > 1. (**B**) CAD mRNA and (**C**) mTORC1 signature score (GSVA; HALLMARK_MTORC1_SIGNALING without RRM2) in primary tumors by GISTIC2 PTEN call: CN-neutral (*n* = 326), shallow deletion (*n* = 64) and deep deletion (*n* = 95). Bars, median, and interquartile range. In (**A**), dashed lines mark the significance thresholds; blue and orange points are significantly down- and up-regulated genes; diamonds mark PTEN, PTENP1 and CAD. In (**B**,**C**), violins show the distribution and points individual tumors. Panel titles give Hedges’ g (95% CI) and Wilcoxon *p*.

Genome-wide tumor-versus-normal differential expression in TCGA-PRAD (Figure 1A) used limma-voom [29,36] on GDC counts (FDR < 0.05, |log_2_FC| > 1). The cross-cohort tumor-versus-normal comparison of CAD used limma on each cohort’s harmonized matrix. Pearson correlations of PTEN with pathway genes were computed in TCGA-PRAD primary tumors (*n* = 497), with all-sample and normal-only values given for comparison (Table S3).

### 2.6. Multivariable Models and Confounders

Ordinary least-squares models were fitted for CAD mRNA, the mTORC1 score and the pyrimidine score, with PTEN deletion as the exposure: M1, unadjusted; M2, plus age, ISUP grade group and ordinal pathologic T stage; M3, plus proliferation; M4, plus AR activity (*n* = 478 with complete covariates; 158 deleted). M5, reported only in the supplementary materials, adds pathologic N stage to M4 in the 409 tumors with a recorded N stage. Model M6 added ABSOLUTE purity, TP53 alteration (mutation or deep deletion), RB1 alteration (mutation or deep deletion), ERG fusion and sequencing batch to M4 in the TCGA 2015 subset with complete data (*n* = 276; 104 deleted), with M4 refitted in the same subset for comparison and M6 without batch as a sensitivity analysis. Post hoc, each added covariate was entered singly into M4, models were fitted within ERG-fusion strata, and, in all 478 tumors, M4 was refitted with ERG mRNA, a proxy for ERG fusion (validated against fusion status in the 2015 subset), as an additional covariate. Deletion depth was assessed by a direct deep-versus-shallow comparison and by an ordinal trend across 0, 1 and 2 lost copies. CAD copy number (GISTIC2) was added to M1 to test whether CAD dosage contributed. The progression-free interval (PFI) of the TCGA Pan-Cancer Clinical Data Resource [26] was analyzed with Cox models for PTEN deletion and per-standard-deviation CAD, mTORC1 and pyrimidine scores, unadjusted and adjusted for grade group and pathologic T stage; median follow-up was estimated by the reverse Kaplan–Meier method.

### 2.7. Non-TCGA Genomic Cohort and Cross-Cohort Meta-Analysis

In the MSKCC cohort, primary tumors with both copy number and expression (*n* = 109) were classified as PTEN-deleted (*n* = 13) or CN-neutral (*n* = 96), signatures were scored in the same way as in TCGA-PRAD, and CAD and both scores were compared, unadjusted and adjusted for Gleason score; the models used the 108 tumors with a Gleason score (one PTEN-deleted tumor lacked one).

Across the eight cohorts, samples were stratified at the within-cohort PTEN mRNA median (ties to PTEN-Low), and the standardized mean difference in CAD mRNA between PTEN-Low and PTEN-High was computed (Hedges’ g). In the principal analysis each cohort was first restricted to primary tumors (k = 8; 538 PTEN-Low, 531 PTEN-High); in a secondary analysis all samples were used. Estimates were pooled with metafor [37], stating both the between-study variance (τ^2^) estimator—DerSimonian–Laird (DL) [38], restricted maximum likelihood (REML) or Paule–Mandel (PM)—and the inference—Wald z or Hartung–Knapp (t with k − 1 df) [39,40]. Leave-one-out analyses were run under both inferences, PTEN–CAD correlations were pooled on the Fisher z scale, and Egger’s test is reported for completeness only [41]. In the post hoc ERG-adjusted version, CAD was regressed in each cohort on PTEN-Low and ERG mRNA (or, as a sensitivity analysis, an ERG-high indicator from a two-cluster split of ERG mRNA), the PTEN-Low coefficient was scaled by the pooled within-group standard deviation of CAD with the small-sample correction, and the cohort estimates were pooled in the same way. The unadjusted estimate from this model-based procedure is reported alongside it. Because ERG adjustment depends on ERG being measured, ERG measurement was checked in each cohort by its dynamic range and by its correlation with CACNA1D, an established ERG-fusion target gene, and with a composite of ERG-fusion target genes (TDRD1, PLA1A, CACNA1D, KCNS3, CRISP3, as available); the ERG-adjusted analysis was repeated without any cohort in which ERG was not measurable.

### 2.8. Pharmacological Control Dataset

GSE138417 comprises MCF7 cells treated with DMSO, MLN0128 or other mTOR inhibitors, with cytosolic and polysome-associated RNA sequenced separately (five replicates per condition). A limma model with a fraction × treatment term and a replicate block was fitted, and MLN0128 versus DMSO was tested in each fraction and for their difference; the pyrimidine set was tested with camera [42].

### 2.9. Statistical Analysis and Reproducibility

All tests were two-sided. Two-group comparisons report Hedges’ g with a 95% confidence interval and the Wilcoxon *p*-value, and FDR control is within predefined families. Analyses used R 4.3.3 (GSVA 1.50.0, limma, metafor 4.4-0, survival) and Python 3.11. Every number, table and figure was generated from the stored data files by the deposited code, and per-sample and per-cohort source data are given in Supplementary Data S1.

## 3. Results

### 3.1. Tumor-Versus-Normal Differential Expression in TCGA-PRAD

Genome-wide, 236 genes were lower and 98 higher in TCGA-PRAD tumors than in normal prostate (Figure 1A). PTEN was the most significantly downregulated gene (log_2_FC = −1.51; FDR = 4.3 × 10^−62^). CAD mRNA was modestly higher in tumor than in normal tissue (log_2_FC = +0.23; FDR = 1.4 × 10^−4^), and core PI3K–AKT–mTOR kinase transcripts changed little.

### 3.2. PTEN Deletion and CAD, mTORC1 and Pyrimidine-Synthesis Expression in TCGA-PRAD (Original Analysis)

**Figure 2:**
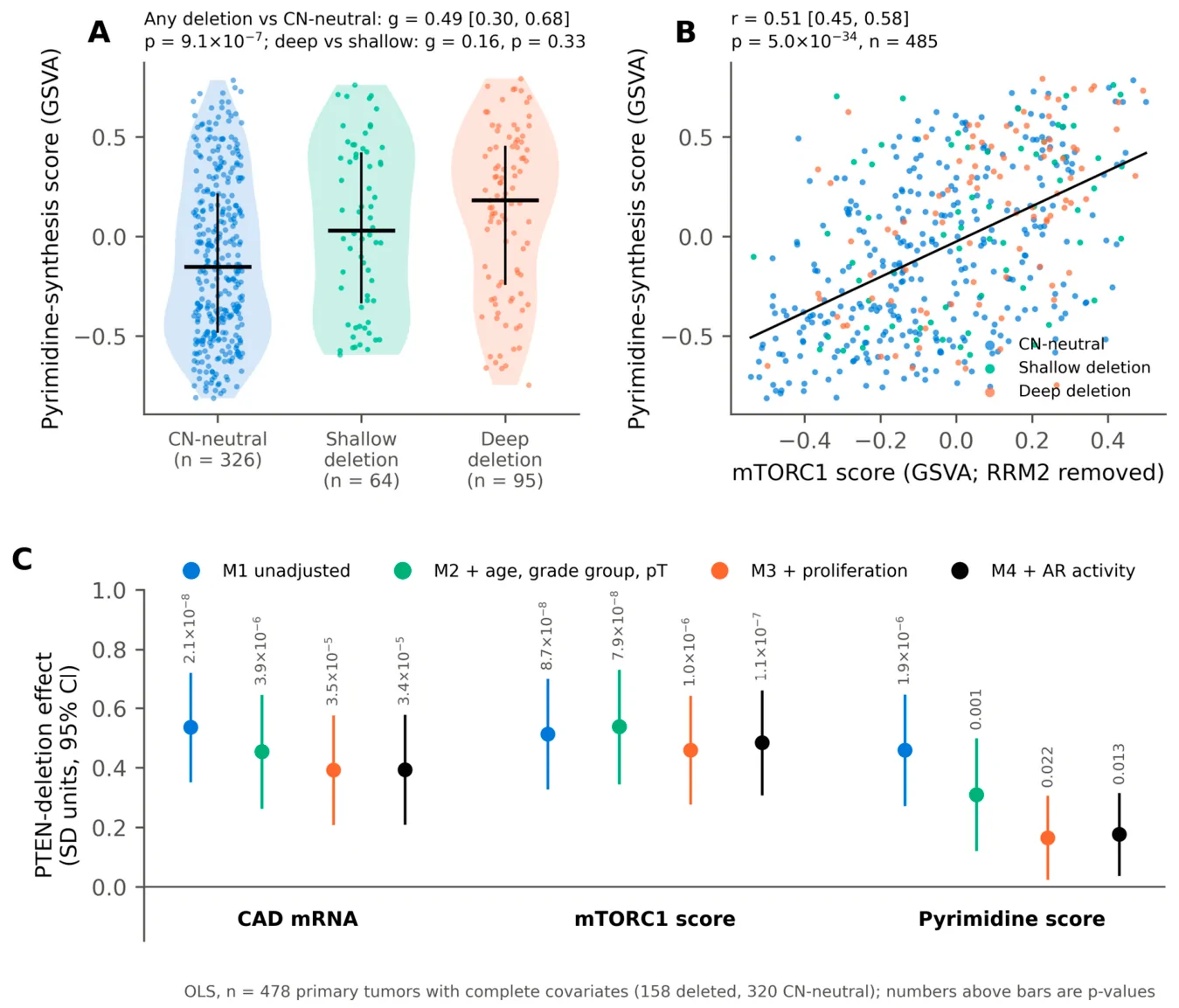
Pyrimidine-synthesis signature and adjustment for clinical and proliferative covariates (TCGA-PRAD). (**A**) Pyrimidine-synthesis score by GISTIC2 PTEN class. (**B**) Pyrimidine-synthesis versus mTORC1 score (*n* = 485; no shared genes). (**C**) Standardized PTEN-deletion effect (β/SD, 95% CI) on CAD mRNA, mTORC1 score and pyrimidine score in models M1–M4 (*n* = 478); numbers are *p*-values. Further adjustment, including for ERG fusion, is shown in Table 3 and Section 3.5.

PTEN-deleted tumors had higher CAD mRNA than CN-neutral tumors (Hedges’ g = 0.57, 95% CI 0.38–0.76; Wilcoxon *p* = 7.9 × 10^−9^), a higher mTORC1 score (g = 0.54, 95% CI 0.35–0.74; *p* = 3.2 × 10^−8^; Figure 1B,C) and a higher pyrimidine-synthesis score (g = 0.49, 95% CI 0.30–0.68; *p* = 9.1 × 10^−7^; Figure 2A; Table 2). The pyrimidine and mTORC1 scores, which share no genes, were correlated (r = 0.51; *p* = 5.0 × 10^−34^; Figure 2B). Effects were numerically larger for deep than for shallow deletion, but deep and shallow deletion did not differ significantly for any outcome (CAD g = 0.26, *p* = 0.17), so no gene-dosage effect is claimed. Within primary tumors, PTEN mRNA was inversely correlated with CAD (r = −0.22; *p* = 1.0 × 10^−6^) and with several pyrimidine-synthesis genes (Figure S1; Table S3).

Adding somatic mutations changed the classification of only 2 tumors: 18 tumors carried a non-silent PTEN mutation, and 16 of them also carried a deletion. All three associations were unchanged under the mutation-aware definition (CAD g = 0.58, *p* = 3.2 × 10^−9^; mTORC1 g = 0.54, *p* = 4.2 × 10^−8^; pyrimidine g = 0.50, *p* = 7.7 × 10^−7^; Table S4), as they were under hybrid copy-number/expression definitions. Copy number at the CAD locus was rarely altered (gain in 17 tumors), and adding it to the model did not change the PTEN coefficient for CAD (β = 0.245 to 0.256; Table S5).

### 3.3. Adjustment for Clinical and Proliferative Covariates (Original Analysis)

PTEN-deleted tumors were more proliferative (proliferation score g = 0.50, *p* = 3.0 × 10^−7^) and of a higher-grade group (*p* = 2.2 × 10^−8^). In models M1–M4 (Table 3; Figure 2C), the PTEN coefficient for CAD fell from 0.236 log_2_ units (*p* = 2.1 × 10^−8^) to 0.173 (95% CI 0.092–0.255; *p* = 3.4 × 10^−5^), and the coefficient for the mTORC1 score was essentially unchanged (standardized effect 0.51 to 0.48; *p* = 1.1 × 10^−7^). The pyrimidine association was largely explained by proliferation (standardized effect 0.46 to 0.18; *p* = 0.013). Complete coefficients are in Table S6.

### 3.4. Protein-Level Readouts (Pre-Specified)

**Figure 3:**
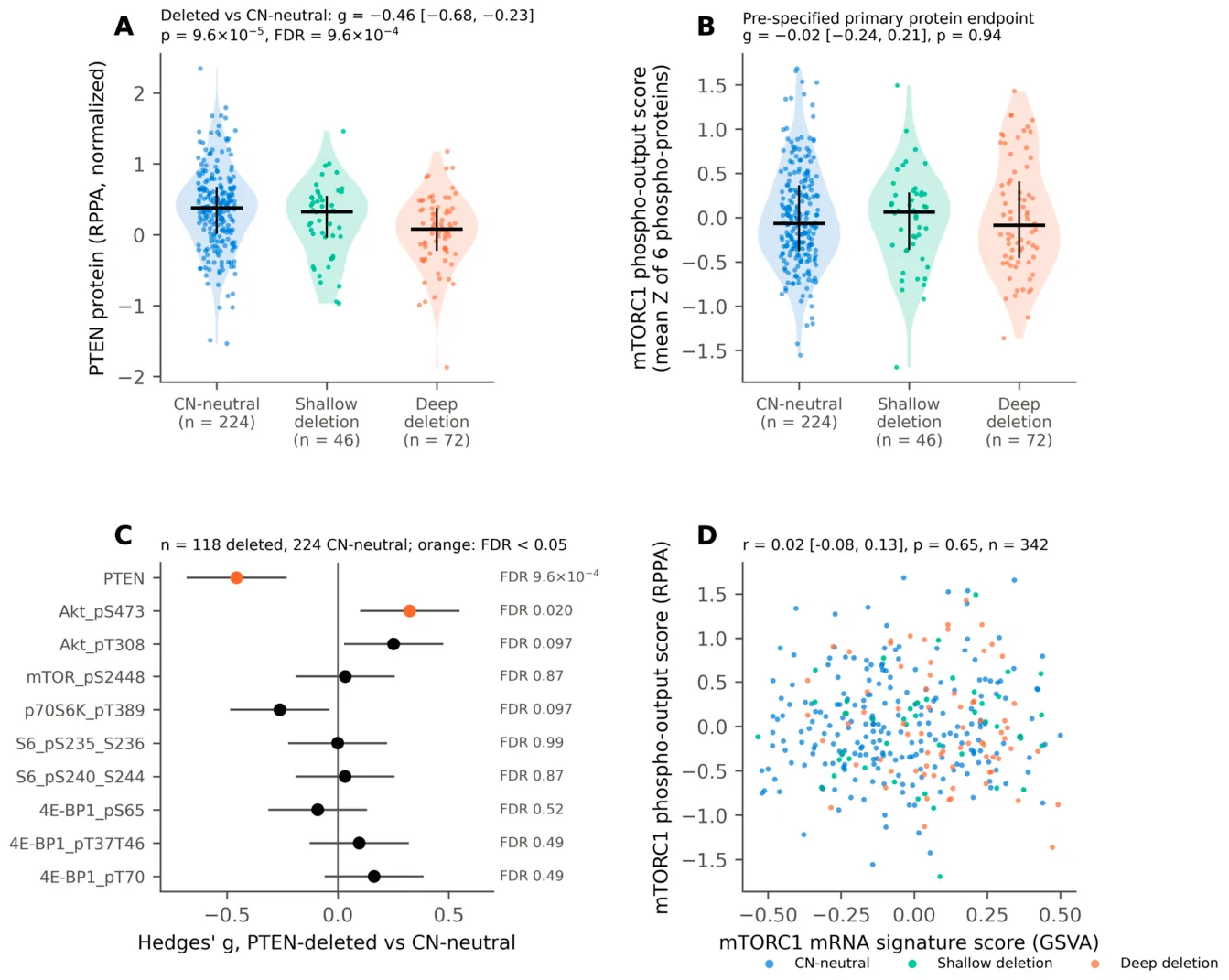
Protein-level readouts by PTEN deletion (TCGA-PRAD RPPA; 342 primary tumors: 224 CN-neutral, 46 shallow and 72 deep deletion). (**A**) PTEN protein. (**B**) Pre-specified primary protein endpoint: mTORC1 phospho-output score (mean Z-score of p70S6K pThr389, S6 pSer235/236, S6 pSer240/244 and 4E-BP1 pSer65, pThr37/46 and pThr70). (**C**) Hedges’ g (95% CI) for each pre-specified antibody, PTEN-deleted versus CN-neutral, with Benjamini–Hochberg FDR within the family; orange, FDR < 0.05. (**D**) Transcriptional mTORC1 score versus the phospho-output score. In (**A**,**B**), violins show the distribution and points individual tumors; horizontal bars mark the median and vertical bars the interquartile range. In (**C**), points are effect sizes and horizontal lines 95% CIs. In (**D**), points are colored by PTEN class (blue, CN-neutral; green, shallow deletion; orange, deep deletion).

In the 342 tumors with RPPA data, PTEN-deleted tumors had lower PTEN protein (g = −0.46, 95% CI −0.68 to −0.23; *p* = 9.6 × 10^−5^; FDR = 9.6 × 10^−4^; Figure 3A,C) and higher AKT pSer473 (g = 0.32, 95% CI 0.10–0.55; FDR = 0.020), with AKT pThr308 higher at nominal significance only (g = 0.25; *p* = 0.039; FDR = 0.097). PTEN mRNA correlated with PTEN protein (r = 0.35, 95% CI 0.25–0.44; *p* = 2.6 × 10^−11^), and the PTEN-protein difference persisted in model M4 (β = −0.24; *p* = 4.3 × 10^−4^). The genomic classification is therefore supported at the protein level, with a moderate effect on PTEN protein and on proximal AKT phosphorylation.

The pre-specified primary protein endpoint, the mTORC1 phospho-output score, did not differ between PTEN-deleted and CN-neutral tumors (g = −0.02, 95% CI −0.24 to 0.21; *p* = 0.94; Figure 3B), including after adjustment (M4 β = 0.045; *p* = 0.51). None of the six output phospho-antibodies was significantly higher in deleted tumors; the S6 and 4E-BP1 sites all had FDR ≥ 0.49. p70S6K pThr389 was nominally lower (g = −0.26, 95% CI −0.49 to −0.04; *p* = 0.036; FDR = 0.097), a direction opposite to the hypothesis that was not significant after FDR correction, and mTOR pSer2448 did not differ (g = 0.03; FDR = 0.87) (Figure 3C). The transcriptional mTORC1 score did not correlate with the phospho-output score (r = 0.02; *p* = 0.65; Figure 3D), and neither did CAD mRNA (r = 0.08; *p* = 0.13). In an exploratory analysis that was not pre-specified, PTEN protein treated as continuous correlated inversely with the phospho-output score (r = −0.25, 95% CI −0.35 to −0.15; *p* = 2.7 × 10^−6^). Across the full panel, 40 of 193 antibodies differed at FDR < 0.05 (Table S7); the pre-specified family results are in Table S8.

These data support the deletion-based PTEN classification at the protein level but provide no evidence that measured mTORC1 output is higher in PTEN-deleted tumors. In line with this, none of the seven alternative mTORC1- or SREBP-related gene sets were associated with PTEN deletion at FDR < 0.05, and one (BILANGES serum- and rapamycin-sensitive genes) was nominally lower (g = −0.22; *p* = 0.015; FDR = 0.10) (Table S9). The association with the Hallmark mTORC1 score is therefore specific to that gene set and should not be read as higher mTORC1 activity.

### 3.5. Adjustment for Genomic Context and the Role of ERG Fusion (Pre-Specified, with Post Hoc Decomposition)

In the TCGA 2015 subset with complete purity, mutation, fusion and batch data (*n* = 276; 104 PTEN-deleted), the pre-specified model M6 removed the PTEN–CAD association (β = −0.002, 95% CI −0.095 to 0.091; *p* = 0.96, compared with β = 0.127, *p* = 0.009 for M4 in the same tumors). The result was the same without batch (β = 0.033; *p* = 0.50). In contrast, the PTEN association with the mTORC1 score was unchanged (M6 β = 0.145, 95% CI 0.086–0.203; *p* = 1.8 × 10^−6^), and the pyrimidine association remained borderline (β = 0.077; *p* = 0.048) (Table 3 and Table S6).

**Figure 4:**
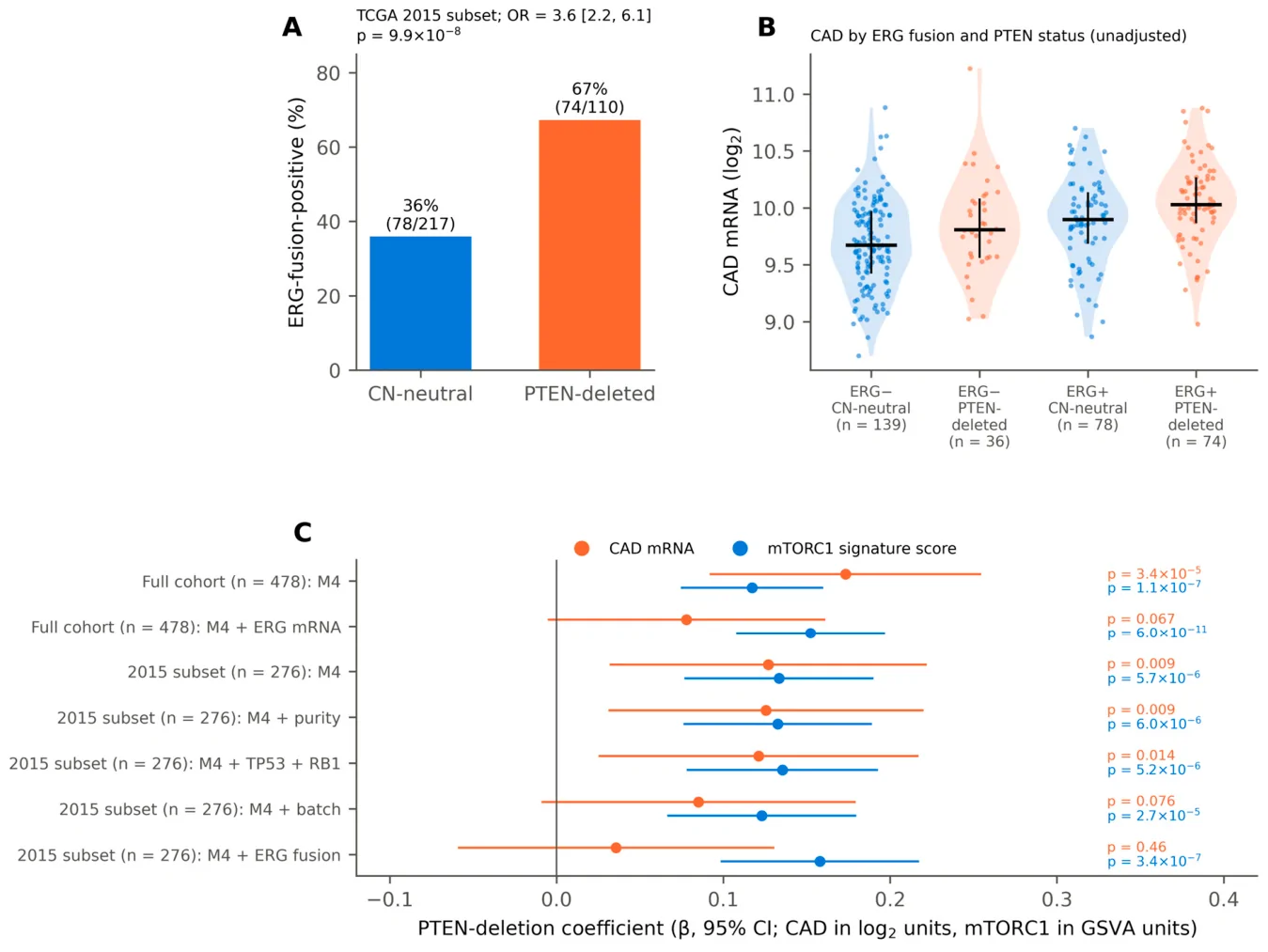
The PTEN–CAD association and ERG fusion. (**A**) Proportion of ERG-fusion-positive tumors by PTEN status (TCGA 2015 subset). (**B**) CAD mRNA by ERG fusion and PTEN status (unadjusted). (**C**) PTEN-deletion coefficient (β, 95% CI) for CAD mRNA (orange) and the mTORC1 score (blue) under successive adjustment: all 478 classified tumors with model M4 and with M4 plus ERG mRNA (post hoc), and the 276 tumors of the 2015 subset with M4 and with M4 plus each additional covariate singly (post hoc decomposition of the pre-specified model M6). Values are in Table 3 and Table S10.

The post hoc decomposition identified ERG fusion as the covariate whose addition changed the coefficient most (Figure 4; Table S10). PTEN-deleted tumors were more often ERG-fusion-positive than CN-neutral tumors (67% versus 36%; odds ratio 3.6, 95% CI 2.2–6.1; *p* = 9.9 × 10^−8^), and ERG-fusion-positive tumors had higher CAD mRNA (g = 0.61; *p* = 2.5 × 10^−8^). Adding ERG fusion alone to M4 reduced the PTEN–CAD coefficient from 0.127 to 0.036 (95% CI −0.059 to 0.131; *p* = 0.46), whereas adding purity (0.126), TP53 and RB1 (0.121), or batch (0.085) changed it much less. Within ERG strata, CAD was higher in PTEN-deleted tumors before adjustment (ERG-negative g = 0.40, *p* = 0.055; ERG-positive g = 0.42, *p* = 0.010) but not after M4 adjustment (ERG-negative β = 0.038, *p* = 0.58; ERG-positive β = 0.027, *p* = 0.64). The mTORC1 association was present in both strata after adjustment (β = 0.150, *p* = 0.0013; β = 0.176, *p* = 2.0 × 10^−6^).

ERG mRNA discriminated ERG-fusion status almost perfectly (area under the curve 0.997 in all 333 tumors of the 2015 study with fusion calls and RNA-seq; 327 of them were classified by PTEN status). Using it as a proxy in all 478 tumors, adding ERG mRNA to M4 reduced the PTEN–CAD coefficient from 0.173 to 0.078 (95% CI −0.005 to 0.161; *p* = 0.067), while the mTORC1 coefficient increased (0.117 to 0.152; *p* = 6.0 × 10^−11^) (Figure 4C; Table 3).

### 3.6. Non-TCGA Genomic Cohort (Pre-Specified)

In the MSKCC cohort (109 primary tumors; 13 PTEN-deleted, of which 6 deep; the same patients as GSE21034), point estimates were in the same direction as in TCGA-PRAD but small and not significant: CAD g = 0.35 (95% CI −0.23 to 0.93; *p* = 0.28), mTORC1 score g = 0.22 (*p* = 0.44), and pyrimidine score g = 0.27 (*p* = 0.38); the Gleason-adjusted CAD coefficient was 0.027 (*p* = 0.69; *n* = 108) (Table S11). With 13 deleted tumors this analysis is underpowered and inconclusive.

### 3.7. Cross-Cohort PTEN mRNA Analysis (Original Analysis), With and Without ERG Adjustment (Post Hoc)

Unstratified tumor-versus-normal CAD differences were highly heterogeneous across the eight cohorts (I^2^ = 97%; pooled *p* = 0.67; Figure S2; Table S12). In the principal PTEN mRNA–stratified analysis of primary tumors, CAD mRNA was modestly higher in PTEN-Low than in PTEN-High tumors (Hedges’ g = 0.227; DL τ^2^ with Wald inference, 95% CI 0.073–0.381, *p* = 0.0039; with Hartung–Knapp inference, 95% CI 0.037–0.417, *p* = 0.026; I^2^ = 21.7%; Table 4). The same contrast run on all samples gave a similar estimate with much greater heterogeneity (g = 0.272; I^2^ = 76.2%; Hartung–Knapp *p* = 0.11; Figure S3; Table S13; GSE21034 sample composition, Table S14). Under Hartung–Knapp inference, the principal estimate lost nominal significance when any one of four cohorts was omitted (Table S15). The PTEN–CAD correlation pooled across primary tumors was r = −0.158 (Hartung–Knapp *p* = 0.023; I^2^ = 45%).

**Figure 5:**
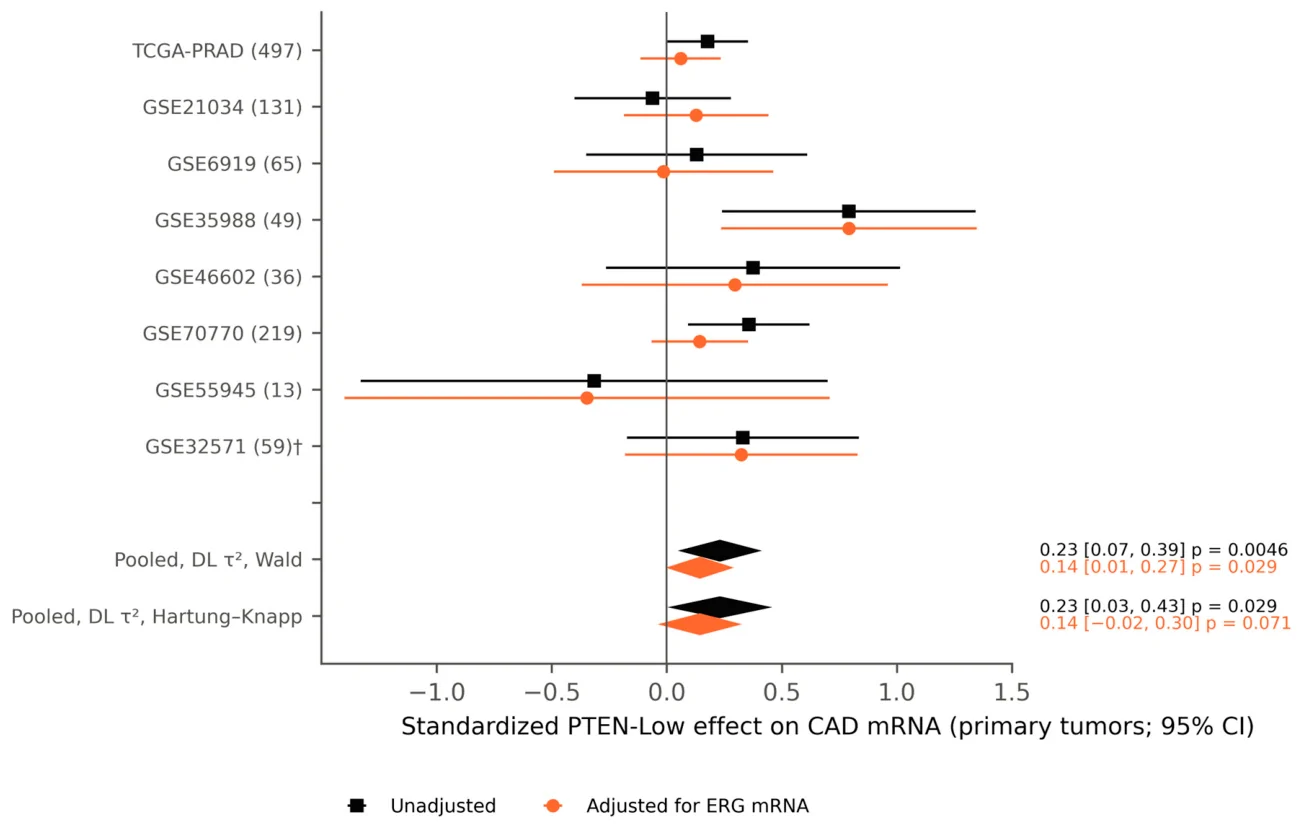
CAD mRNA in PTEN-Low versus PTEN-High primary tumors across eight cohorts, unadjusted (black; model-based version of the principal analysis) and adjusted for ERG mRNA (orange; post hoc). Per-cohort standardized effects (95% CI; *n* primary tumors in parentheses) and pooled estimates with DerSimonian–Laird τ^2^ under Wald and Hartung–Knapp inference; values at right. Both estimates are model-based so that they are directly comparable (Table 4). † ERG was not measurable in GSE32571 (probes at background), so its ERG-adjusted estimate is uninformative; the pooled ERG-adjusted estimate without it is in Table S16.

PTEN mRNA was inversely correlated with ERG mRNA in the primary tumors of seven of eight cohorts (r between −0.40 and −0.13). Adjustment for ERG mRNA reduced the pooled PTEN-Low effect from 0.231 (model-based unadjusted estimate; Hartung–Knapp *p* = 0.029) to 0.144 (95% CI 0.015–0.273, Wald *p* = 0.029; Hartung–Knapp 95% CI −0.016 to 0.304, *p* = 0.071; I^2^ = 12.7%, against 27.2% for the model-based unadjusted estimate), and adjustment for an ERG-high indicator gave 0.162 (Hartung–Knapp *p* = 0.049) (Figure 5; Table 4). ERG mRNA was measurable and tracked the ERG-fusion target CACNA1D (r = 0.42 to 0.87), in seven cohorts. In GSE32571 (Illumina HumanHT-12 v3), all three ERG probes were close to the array background (ERG interquartile range 0.12 log_2_ units, against 0.41–5.3 elsewhere), ERG did not correlate with CACNA1D (r = −0.01) or with the target-gene composite, and the two-cluster split classified only 2 of 59 tumors (3%) as ERG-high, against 31–63% in the other cohorts (Table S16). ERG adjustment is therefore uninformative in that cohort. Excluding it gave an ERG-adjusted estimate of 0.136 (Hartung–Knapp 95% CI −0.051 to 0.323, *p* = 0.12; unadjusted 0.224, Hartung–Knapp *p* = 0.062). In GSE21034, where ERG tracked CACNA1D (r = 0.87) but not the target-gene composite (r = 0.07), the estimate changed sign after adjustment (−0.061 unadjusted, 0.128 adjusted); the confidence intervals of both estimates were wide. ERG adjustment therefore attenuated the cross-cohort estimate by about 38%, with widely overlapping confidence intervals; the adjusted estimate is not significant under Hartung–Knapp inference and reaches nominal significance only under Wald inference. Without GSE32571 the unadjusted estimate is itself not significant under Hartung–Knapp inference (*p* = 0.062), so that sensitivity analysis does not by itself show an effect of adjustment.

### 3.8. Progression-Free Interval (Pre-Specified) and Pharmacological Control (Original Analysis)

PTEN deletion (HR 1.56, 95% CI 1.03–2.36; *p* = 0.037), CAD mRNA (HR 1.44 per SD; *p* = 9.2 × 10^−4^) and the pyrimidine score (HR 1.49 per SD; *p* = 2.7 × 10^−4^) were associated with a shorter progression-free interval in unadjusted models (482 tumors; 90 events; median follow-up 30.9 months), but none remained associated after adjustment for grade group and pathologic T stage (HR 1.02, 1.18 and 1.16; all *p* > 0.1); the mTORC1 score was not associated in either model (Table S17). In MCF7 cells, MLN0128 lowered pyrimidine-synthesis transcripts in both cytosolic RNA (camera *p* = 4.3 × 10^−4^) and polysome-associated RNA (*p* = 1.7 × 10^−3^), with no translational component (*p* = 0.98), whereas the 5′TOP control RPS6 showed the expected loss from polysomes [43]; CAD did not change (Figure S4; Table S18).

## 4. Discussion

This study set out to test whether PTEN loss in prostate cancer is associated with CAD expression and with mTORC1 and pyrimidine-synthesis programs. It used public data at three levels and confronted each transcript-level finding with protein measurements and with the genomic context of the tumors. Three findings emerge; the second and third qualify the original hypothesis.

First, the genomic PTEN classification is supported at the protein level. PTEN-deleted tumors had lower PTEN protein and higher AKT Ser473 phosphorylation, and PTEN mRNA tracked PTEN protein. This supports the use of GISTIC2 deletion as a PTEN-loss classifier in TCGA-PRAD and is consistent with the established PI3K–AKT biology of PTEN [5,6].

Second, the association of PTEN deletion with the Hallmark mTORC1 transcriptional score is robust to grade, stage, proliferation, AR activity, purity, TP53, RB1, ERG fusion, and batch, but it is not accompanied by higher measured mTORC1 output. Neither S6K1, S6 nor 4E-BP1 phosphorylation was significantly higher in PTEN-deleted tumors, the transcriptional score did not correlate with the phospho-output composite, and other mTORC1-related gene sets were not associated with PTEN deletion. Several explanations are possible. RPPA measures bulk tumor lysates at one time point, phosphorylation is labile after resection, mTORC1 integrates inputs other than PTEN–AKT [13], and the Hallmark set contains biosynthetic genes that respond to other transcriptional programs [44]. Whatever the reason, the transcriptional score should not be described as mTORC1 activity in these tumors. The exploratory inverse correlation between continuous PTEN protein and the phospho-output score suggests that PTEN protein level may relate to mTORC1 output more closely than copy number does, and this could be examined in cohorts with richer protein data.

Third, in these data the association between PTEN deletion and CAD mRNA is not independent of ERG fusion together with grade, stage, and proliferation. PTEN loss is enriched in ERG-fusion-positive tumors, as reported previously [18,19], and here ERG-fusion tumors had higher CAD mRNA. We treated ERG fusion as a common cause rather than a mediator: TMPRSS2–ERG fusion is generally an early, often truncal event, and in tumors that carry both alterations ERG fusion precedes PTEN deletion [45]. Had ERG fusion lain downstream of PTEN loss, adjustment would have removed part of a real effect; these data cannot test the order of events in individual tumors. Of the covariates added, ERG fusion changed the PTEN coefficient most, but it is not a complete explanation on its own: within ERG strata, CAD was still higher in PTEN-deleted tumors before adjustment (g ≈ 0.4), and the association disappeared only when the clinical and proliferation covariates were added within strata. With these adjustments the PTEN–CAD association was no longer detectable in TCGA-PRAD, and the cross-cohort PTEN-Low effect was attenuated by about 38%, with overlapping confidence intervals, to a small estimate that was not significant under Hartung–Knapp inference. The original question—whether PTEN loss increases CAD expression through the mTORC1–S6K1 axis—therefore receives no support from these data. They do not exclude an effect on CAD phosphorylation, which was not measured, or a small ERG-independent effect below the resolution of these cohorts. The pyrimidine-synthesis signature behaved like a proliferation marker, and the unadjusted survival associations of PTEN deletion, CAD and the pyrimidine score did not survive adjustment for grade and stage.

These results carry a general lesson for transcriptomic pathway analyses of molecularly heterogeneous tumors. An association between a tumor suppressor and a downstream transcript can arise from co-occurring genomic events, and a pathway “activity” signature can be strongly associated with a genotype without any corresponding difference in measured pathway output. Protein-level checks and adjustment for co-occurring driver events, where they are available, can change the interpretation substantially, as they did here.

### 4.1. Limitations

This is an observational, exploratory analysis of public data, and it cannot establish causation. Protein data came from RPPA, which does not include CAD or pCAD(Ser1859), so the question of whether PTEN loss changes CAD phosphorylation in patient tumors remains open. The cell-line Western blots in Supplementary Note S1 (Figures S5–S7) are single experiments without viability controls or PTEN measurement, in a series that is not a PTEN-loss model, and do not contribute to the conclusions. Experimental validation is outside the scope of this study. Fusion, purity, and batch data were available only for the TCGA 2015 subset, and ERG mRNA served as a proxy elsewhere; the ERG analyses were added after the confounding result was known and are labeled post hoc. The analyzed subsets were not random. The RPPA subset (342 of 485 tumors) had more pT3–T4 tumors than the remainder (68% versus 45%), and the M6 subset (276 tumors) had a higher proportion of PTEN deletion (38% versus 26%) and higher CAD mRNA than the tumors outside it, and the TCGA 2015 study tumors differed slightly in grade group from the remainder (*p* = 0.044) (Table S19). The non-TCGA genomic cohort contained only 13 PTEN-deleted tumors and shares its patients with GSE21034. ERG was not measurable in one cohort (GSE32571). The cross-cohort analysis uses a PTEN mRNA proxy, and its principal-analysis designation was made post hoc. The proteomic cohort of Sinha et al. and cell-line dependency data could not be obtained for this study.

### 4.2. Future Directions

Whether PTEN loss changes CAD phosphorylation or pyrimidine flux in prostate cancer independently of ERG fusion would be tested most directly by measuring pCAD(Ser1859) and isotope-traced pyrimidine synthesis in isogenic PTEN-proficient and PTEN-deficient prostate models with and without ERG expression. Prostate tumor cohorts with mass-spectrometry proteomics and fusion status would allow CAD protein to be examined in patients. These experiments are beyond the scope of the present analysis.

## 5. Conclusions

In primary prostate cancer, PTEN deletion is reflected in lower PTEN protein and higher AKT Ser473 phosphorylation, and it is robustly associated with the Hallmark mTORC1 transcriptional signature, but not with measured mTORC1 phospho-outputs. In TCGA-PRAD, the association between PTEN deletion and CAD mRNA is not independent of co-occurring ERG fusion together with grade, stage, and proliferation, with ERG fusion the covariate whose addition changes the association most, and the cross-cohort PTEN mRNA–CAD association is attenuated after ERG adjustment to a small estimate that is not significant under Hartung–Knapp inference. CAD protein, CAD phosphorylation and pyrimidine flux were not measured, and these findings carry no implication for clinical actionability.

## Figures and Tables

**Table 1:** Cohorts, platforms, and the PTEN-related data used.

Cohort	Platform	*n* (Tumor/Normal)	Disease Setting	PTEN and Related Data Used	PTEN-Low/High, All Samples	PTEN-Low/High, Primary	Clinical Metadata
TCGA-PRAD	Illumina HiSeq 2000 RNA-seq	550 (498/52)	497 primary + 1 metastasis	GISTIC2 copy number, MC3 mutations, RPPA protein, mRNA; ERG fusion, purity, batch in 333 (TCGA 2015)	275/275	249/248	Gleason/ISUP grade, pT, pN, age, PFI
GSE21034	Affymetrix Human Exon 1.0 ST	185 (156/29)	131 primary + 19 metastasis + 6 cell line/xenograft	mRNA (copy number for the same patients via the MSKCC cBioPortal study)	93/92	66/65	Gleason, clinical and pathological stage
GSE6919	Affymetrix HG-U95Av2	171 (90/81)	65 primary + 25 metastasis; donor and adjacent normal	mRNA only	86/85	33/32	Tissue type, age, race, stage, Gleason
GSE35988	Agilent G4502A	88 (76/12)	49 primary + 27 metastatic CRPC	mRNA only	44/44	25/24	Sample type only
GSE46602	Affymetrix HG-U133 Plus 2	50 (36/14)	36 primary	mRNA only	25/25	18/18	Gleason, pT, margin, PSA, BCR, age
GSE70770	Illumina HumanHT-12 v4	293 (219/74)	219 primary	mRNA only	147/146	110/109	Gleason, cellularity, PSA, age
GSE55945	Affymetrix HG-U133 Plus 2	21 (13/8)	13 primary	mRNA only	11/10	7/6	Tissue type, TMPRSS2-ERG
GSE32571	Illumina HumanHT-12 v3	98 (59/39)	59 primary, 39 benign	mRNA only	49/49	30/29	Gleason pattern group

Totals: 730/726 PTEN-Low/High in the all-sample analysis (*n* = 1456) and 538/531 in the principal primary tumor analysis (*n* = 1069). “mRNA only”: no PTEN copy-number, mutation or protein data exist for that cohort. For TCGA-PRAD, ERG fusion, purity and batch come from the 333 tumors of the TCGA 2015 study; 327 of these have a PTEN classification, and 276 have complete data for model M6. CRPC, castration-resistant prostate cancer; BCR, biochemical recurrence; PFI, progression-free interval (TCGA Pan-Cancer Clinical Data Resource).

**Table 2:** PTEN deletion and CAD mRNA, mTORC1 score and pyrimidine-synthesis score in TCGA-PRAD primary tumors (CN-neutral *n* = 326; deep deletion *n* = 95; shallow deletion *n* = 64).

Outcome	Contrast	*n*	Means	Hedges’ g (95% CI)	Wilcoxon *p*
CAD mRNA	Any deletion vs. CN-neutral	159 vs. 326	9.908 vs. 9.663	0.57 (0.38 to 0.76)	7.9 × 10^−9^
CAD mRNA	Deep (−2) vs. CN-neutral	95 vs. 326	9.950 vs. 9.663	0.67 (0.43 to 0.90)	1.6 × 10^−8^
CAD mRNA	Shallow (−1) vs. CN-neutral	64 vs. 326	9.845 vs. 9.663	0.42 (0.15 to 0.69)	0.0028
CAD mRNA	Deep vs. shallow	95 vs. 64	9.950 vs. 9.845	0.26 (−0.06 to 0.57)	0.17
CAD mRNA	Ordinal trend, 0/1/2 copies lost	485	—	0.33 SD per copy	2.7 × 10^−9^ (trend)
mTORC1 score	Any deletion vs. CN-neutral	159 vs. 326	0.062 vs. −0.067	0.54 (0.35 to 0.74)	3.2 × 10^−8^
mTORC1 score	Deep (−2) vs. CN-neutral	95 vs. 326	0.076 vs. −0.067	0.61 (0.37 to 0.84)	2.3 × 10^−7^
mTORC1 score	Shallow (−1) vs. CN-neutral	64 vs. 326	0.040 vs. −0.067	0.45 (0.18 to 0.72)	0.0015
mTORC1 score	Deep vs. shallow	95 vs. 64	0.076 vs. 0.040	0.16 (−0.16 to 0.47)	0.36
mTORC1 score	Ordinal trend, 0/1/2 copies lost	485	—	0.31 SD per copy	3.7 × 10^−8^ (trend)
Pyrimidine score	Any deletion vs. CN-neutral	159 vs. 326	0.089 vs. −0.114	0.49 (0.30 to 0.68)	9.1 × 10^−7^
Pyrimidine score	Deep (−2) vs. CN-neutral	95 vs. 326	0.116 vs. −0.114	0.56 (0.33 to 0.79)	3.6 × 10^−6^
Pyrimidine score	Shallow (−1) vs. CN-neutral	64 vs. 326	0.048 vs. −0.114	0.39 (0.13 to 0.66)	0.0057
Pyrimidine score	Deep vs. shallow	95 vs. 64	0.116 vs. 0.048	0.16 (−0.15 to 0.48)	0.33
Pyrimidine score	Ordinal trend, 0/1/2 copies lost	485	—	0.28 SD per copy	4.0 × 10^−7^ (trend)

Hedges’ g compares the first group with the second. The trend row is the coefficient from regressing the standardized outcome on the number of lost copies. The significant trend mainly reflects the difference between any deletion and none: deep and shallow deletion did not differ significantly for any outcome, so no gene-dosage effect is inferred. Mutation-aware and alternative definitions are in Table S4.

**Table 3:** PTEN-deletion coefficients for CAD mRNA, mTORC1 score, and pyrimidine-synthesis score under successive adjustment (TCGA-PRAD primary tumors).

Outcome	Model	Analysis	*n*	β, PTEN Deletion (95% CI)	*p*
CAD mRNA	M1, all classified tumors	original	478	0.236 (0.155 to 0.318)	2.1 × 10^−8^
CAD mRNA	M4, all classified tumors	original	478	0.173 (0.092 to 0.255)	3.4 × 10^−5^
CAD mRNA	M4 + ERG mRNA, all classified tumors	post hoc	478	0.078 (−0.005 to 0.161)	0.067
CAD mRNA	M4, TCGA 2015 subset	pre-specified	276	0.127 (0.032 to 0.222)	0.0090
CAD mRNA	M6 (M4 + purity, TP53, RB1, ERG fusion, batch)	pre-specified	276	−0.002 (−0.095 to 0.091)	0.96
CAD mRNA	M4 + ERG fusion, TCGA 2015 subset	post hoc	276	0.036 (−0.059 to 0.131)	0.46
mTORC1 score	M1, all classified tumors	original	478	0.124 (0.079 to 0.169)	8.7 × 10^−8^
mTORC1 score	M4, all classified tumors	original	478	0.117 (0.075 to 0.160)	1.1 × 10^−7^
mTORC1 score	M4 + ERG mRNA, all classified tumors	post hoc	478	0.152 (0.108 to 0.197)	6.0 × 10^−11^
mTORC1 score	M4, TCGA 2015 subset	pre-specified	276	0.133 (0.077 to 0.190)	5.7 × 10^−6^
mTORC1 score	M6 (M4 + purity, TP53, RB1, ERG fusion, batch)	pre-specified	276	0.145 (0.086 to 0.203)	1.8 × 10^−6^
mTORC1 score	M4 + ERG fusion, TCGA 2015 subset	post hoc	276	0.158 (0.098 to 0.217)	3.4 × 10^−7^
Pyrimidine score	M1, all classified tumors	original	478	0.193 (0.115 to 0.272)	1.9 × 10^−6^
Pyrimidine score	M4, all classified tumors	original	478	0.074 (0.016 to 0.133)	0.013
Pyrimidine score	M4 + ERG mRNA, all classified tumors	post hoc	478	0.087 (0.025 to 0.150)	0.0065
Pyrimidine score	M4, TCGA 2015 subset	pre-specified	276	0.070 (−0.002 to 0.141)	0.055
Pyrimidine score	M6 (M4 + purity, TP53, RB1, ERG fusion, batch)	pre-specified	276	0.077 (0.001 to 0.153)	0.048
Pyrimidine score	M4 + ERG fusion, TCGA 2015 subset	post hoc	276	0.063 (−0.012 to 0.139)	0.100

M1, unadjusted; M4, adjusted for age, ISUP grade group, ordinal pT, proliferation score and AR-activity score. All classified tumors: *n* = 478 (158 PTEN-deleted). TCGA 2015 subset with complete purity, mutation, fusion, and batch data: *n* = 276 (104 PTEN-deleted). β is in log_2_ units for CAD and GSVA units for the scores. Further models and all covariate coefficients are in Tables S6 and S10.

**Table 4:** CAD mRNA in PTEN-Low versus PTEN-High primary tumors across eight cohorts, unadjusted (principal analysis) and adjusted for ERG mRNA (post hoc).

Cohort	*n* Primary	Low/High	Hedges’ g, Unadjusted (95% CI)	*p*	r(PTEN, ERG)	ERG-High (%)	ERG-mRNA-Adjusted Effect (95% CI)
TCGA-PRAD	497	249/248	0.178 (0.00 to 0.35)	0.048	−0.27	40	0.060 (−0.11 to 0.23)
GSE21034	131	66/65	−0.061 (−0.40 to 0.28)	0.73	−0.14	56	0.128 (−0.19 to 0.44)
GSE6919	65	33/32	0.130 (−0.36 to 0.62)	0.60	−0.28	31	−0.014 (−0.49 to 0.46)
GSE35988	49	25/24	0.791 (0.21 to 1.37)	0.0079	−0.19	39	0.792 (0.24 to 1.35)
GSE46602	36	18/18	0.375 (−0.28 to 1.03)	0.26	−0.40	58	0.297 (−0.37 to 0.96)
GSE70770	219	110/109	0.357 (0.09 to 0.62)	0.0087	−0.14	63	0.144 (−0.07 to 0.35)
GSE55945	13	7/6	−0.315 (−1.41 to 0.78)	0.55	−0.13	54	−0.346 (−1.40 to 0.71)
GSE32571	59	30/29	0.330 (−0.18 to 0.84)	0.20	0.05	3	0.324 (−0.18 to 0.83)
Pooled, fixed effect	1069	538/531	0.217 (0.097 to 0.338)	4.2 × 10^−4^			
Pooled, DL τ^2^, Wald	1069	538/531	0.227 (0.073 to 0.381)	0.0039			
Pooled, DL τ^2^, Hartung–Knapp	1069	538/531	0.227 (0.037 to 0.417)	0.026			
Pooled, REML τ^2^, Hartung–Knapp	1069	538/531	0.223 (0.042 to 0.404)	0.023			
Model-based, DL τ^2^, Wald	1069	538/531	0.231 (0.071 to 0.391); *p* = 0.0046				0.144 (0.015 to 0.273); *p* = 0.029
Model-based, DL τ^2^, Hartung–Knapp	1069	538/531	0.231 (0.032 to 0.430); *p* = 0.029				0.144 (−0.016 to 0.304); *p* = 0.071

Unadjusted heterogeneity (DL): I^2^ = 21.7%, τ^2^ = 0.010, Q_7_ = 8.94 (*p* = 0.26); 95% prediction interval −0.02 to 0.48. ERG-adjusted heterogeneity: I^2^ = 12.7%. The ERG-adjusted effect is the PTEN-Low coefficient from a within-cohort regression on PTEN-Low and ERG mRNA, scaled by the pooled within-group SD of CAD; the model-based unadjusted estimate is shown beside it for direct comparison (it differs slightly from the large-sample estimate because its SE comes from the regression). Adjustment for an ERG-high indicator (two-cluster split of ERG mRNA; ERG-high column) gave 0.162 (Hartung–Knapp *p* = 0.049). ERG was not measurable in GSE32571 (probes at background; Table S16); without GSE32571 the ERG-adjusted estimate was 0.136 (Hartung–Knapp 95% CI −0.051 to 0.323; *p* = 0.12). Leave-one-out and correlation analyses are in Table S15; the all-sample analysis is in Table S13.

## Data Availability

All data are publicly available from GEO (GSE6919, GSE21034, GSE32571, GSE35988, GSE46602, GSE55945, GSE70770, GSE138417), the UCSC Xena TCGA hub, the Genomic Data Commons and cBioPortal (studies prad_tcga_pan_can_atlas_2018, prad_tcga_pub and prad_mskcc). Analysis code, the pre-specified analysis plan (commit d0118c8, 3 October 2026) and its addendum (commit 9830705, 3 October 2026), a data manifest with checksums, the erratum, the source data (Supplementary Data S1) and the original, uncropped Western blot image files (also shown in Figure S7) are available at https://github.com/itika05/pten-mtorc1-cad-prostate (release v2.0-resubmission; accessed on 29 October 2026).

## Supplementary Materials

The following supporting information can be downloaded at: https://www.mdpi.com/article/10.3390/cells15211997/s1, Figure S1: PTEN mRNA correlations within TCGA-PRAD primary tumors. (A) CAD versus PTEN mRNA, colored by GISTIC2 class. (B) Correlation of each pathway gene with PTEN in primary tumors (filled, FDR < 0.05) and across all samples (orange tick). Values in Table S3. Figure S2: Cross-cohort context. (A) CAD log_2_ fold change, tumor versus normal, per cohort with the REML pooled estimate. (B) PTEN–CAD correlations per cohort across all samples (orange) and in primary tumors (black), with pooled estimates. Values in Tables S12 and S15. Figure S3: Effect of sample composition: Hedges’ g for CAD mRNA, PTEN-Low versus PTEN-High, in primary tumors only (black) and in all samples (orange), with pooled estimates under three estimator/inference combinations. Values in Table 4 and Table S13. Figure S4: Response of de novo pyrimidine-synthesis and mTORC1/translation transcripts to MLN0128 in MCF7 cells (GSE138417), by RNA fraction: cytosolic (blue), polysome-associated (orange) and their difference (green); dots, FDR < 0.05. Values in Table S18. Table S1: Gene lists. Table S2: Sensitivity of signature results to scoring method (TCGA-PRAD; 159 deleted vs. 326 CN-neutral). Table S3: Pearson correlation of PTEN mRNA with pathway-gene mRNA in TCGA-PRAD. Table S4: Robustness of the TCGA-PRAD associations to the PTEN definition, including the pre-specified mutation-aware definition. Table S5: CAD locus copy number (GISTIC2) in TCGA-PRAD. Table S6: Complete coefficients for multivariable models M1–M5 (all classified tumors) and M6 (TCGA 2015 subset). Table S7: Exploratory: all RPPA antibodies, PTEN-deleted versus CN-neutral (193 antibodies with data). Table S8: Pre-specified protein-level analysis (TCGA-PRAD RPPA; 118 PTEN-deleted, 224 CN-neutral). Table S9: Alternative mTORC1- and SREBP-related gene sets (MSigDB c2), PTEN-deleted (*n* = 159) versus CN-neutral (*n* = 326). Table S10: Post hoc ERG analyses. Table S11: Non-TCGA genomic cohort: MSKCC (Taylor et al.; same patients as GSE21034), primary tumors with copy number and expression (13 PTEN-deleted, of which 6 deep; 96 CN-neutral; models use the 108 tumors with a Gleason score). Table S12: CAD tumor-versus-normal differential expression by cohort (limma; all tumor samples versus normal; cell lines and xenografts excluded). Table S13: Secondary, composition-mixed analysis: CAD mRNA in PTEN-Low versus PTEN-High samples using all samples in each cohort. Table S14: GSE21034 sample composition by PTEN stratum (single median split across all 185 samples). Table S15: Robustness of the principal cross-cohort analysis. Table S16: ERG measurement by cohort (primary tumors) and ERG-adjusted cross-cohort analysis without GSE32571 (post hoc). Table S17: Progression-free interval (TCGA Pan-Cancer Clinical Data Resource PFI; Cox models; pre-specified, exploratory; median follow-up 30.9 months, 95% CI 28.6–34.9, reverse Kaplan–Meier). Table S18: GSE138417 (MCF7): MLN0128 versus DMSO by RNA fraction. Table S19: Characteristics of the analyzed TCGA-PRAD subsets compared with all classified primary tumors (post hoc, descriptive). Supplementary Note S1: Exploratory Cell-Line Western Blots (Single Experiments; Not Used for Any Conclusion of the Main Manuscript). Figure S5: Exploratory baseline Western blot (single experiment; Supplementary Note S1). pCAD (Ser1859), total CAD, phospho-S6K1 (Thr389), total S6K1 and β-actin in the parental RWPE-1 line and four WPE1 derivatives (NA22, NB14, NB11, NB26). Each strip is a single continuous rectangular region of the original image file (outlined in Figure S7A–D), scaled uniformly, without splicing or brightness/contrast adjustment; grey levels are those of the original files, and strips are enlarged about 3–4-fold relative to Figure S7. Phospho- and total S6K1 are on separate membranes. PTEN was not measured; the series is not a PTEN-loss model. Figure S6: Exploratory fisetin treatment (single experiment per line; Supplementary Note S1). pCAD (Ser1859), total CAD and β-actin in WPE1-NB26 and WPE1-NB11 treated with fisetin (0, 20, 40, 60 µM, 48 h). Each line is shown with its own β-actin (Figure S7G). Strips are continuous regions of the original image files (outlined in Figure S7E–G), scaled uniformly, without splicing or brightness/contrast adjustment; grey levels are those of the original files, and strips are enlarged relative to Figure S7. β-actin and total CAD vary between lanes. In WPE1-NB11, β-actin is very weak at 40 µM and weaker at 60 µM than at 0–20 µM, in the lanes with the weakest pCAD signal. No densitometry or statistical comparison is reported. Cell viability was not assessed. Figure S7: Original, uncropped Western blot image files for Figures S5 and S6. (A) pCAD (Ser1859), upper, and total CAD, lower: two separate membranes imaged together (baseline panel); (B) total S6K1; (C) phospho-S6K1 (Thr389); (D) β-actin, with molecular-weight ladder; (E) WPE1-NB26, pCAD (Ser1859) left and total CAD right; (F) WPE1-NB11, pCAD (Ser1859) left and total CAD right; (G) β-actin for WPE1-NB26 (left membrane) and WPE1-NB11 (right membrane). Red rectangles, added here, mark the regions shown in Figures S5 and S6; the image content is otherwise unaltered. Lanes outside the rectangles in (A) and (D) are not part of this study. Molecular-weight markers are visible only in (D)–(G). The files are low-resolution exports without acquisition metadata. Supplementary Data S1: per-sample and per-cohort source data for every figure and table (Excel workbook; the README sheet lists the contents). References [46,47,48,49,50,51,52] are cited in the Supplementary Materials.
