# Supplementary material for: PTEN Deletion, mTORC1 Signatures and CAD Expression in Primary Prostate Cancer: Protein-Level Data and the Role of ERG Fusion
Source: Cells. 2026 Oct 31;15(21):1997. doi: 10.3390/cells15211997 (PMC13761547; doi:10.3390/cells15211997)
Supplement: Supplementary file 1 [file cells-15-01997-s001.zip › 3_Supplementary_Materials_v7_IA.pdf]

cells

MDPI

Supplementary Materials

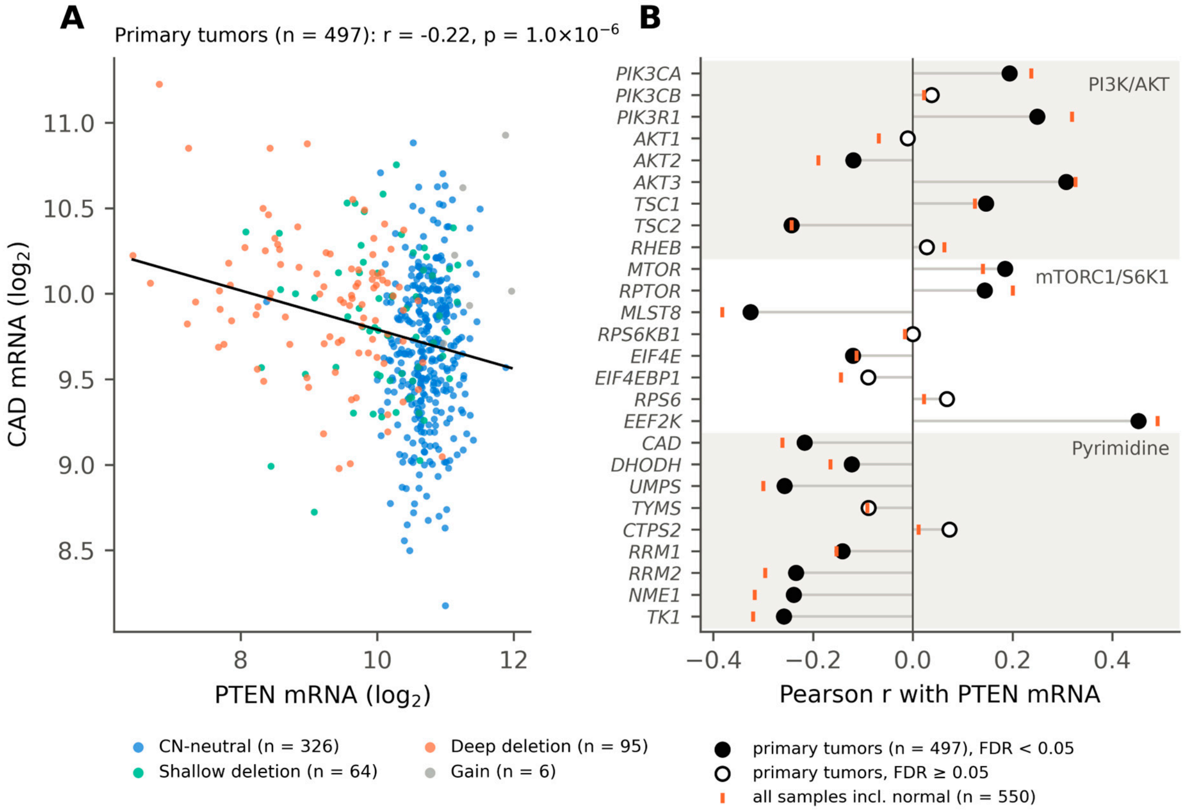

Figure S1. PTEN mRNA correlations within TCGA-PRAD primary tumors. (A) CAD versus PTEN mRNA, colored by GISTIC2 class. (B) Correlation of each pathway gene with PTEN in primary tumors (filled, FDR < 0.05) and across all samples (orange tick). Values in Table S3.

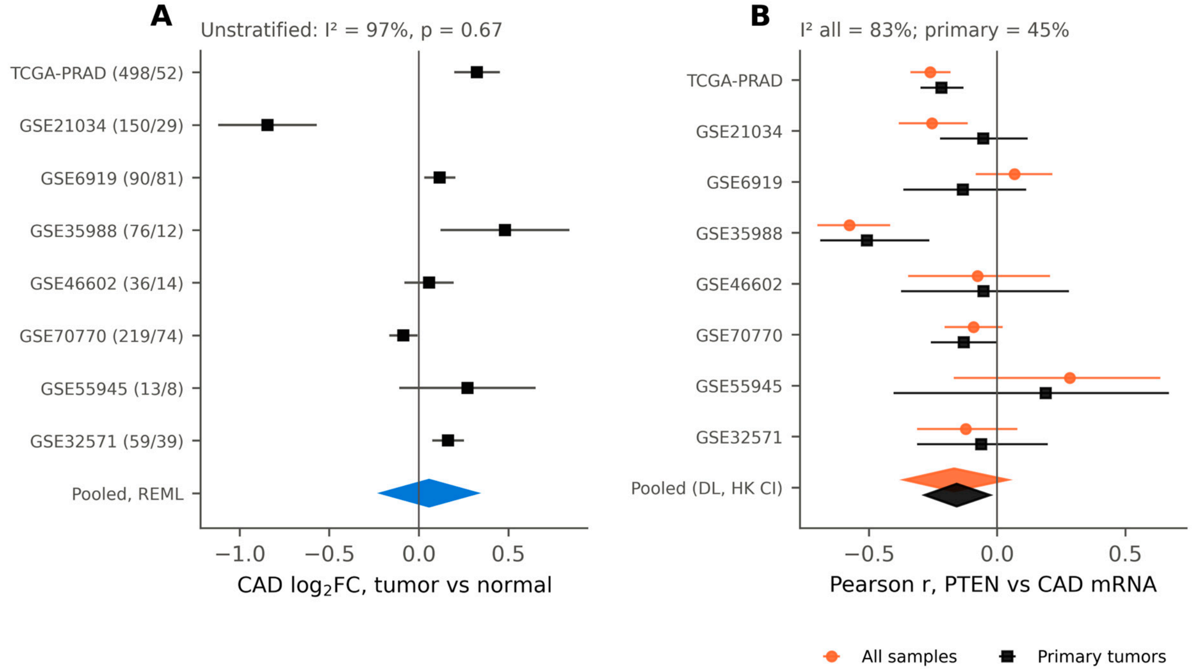

Figure S2. Cross-cohort context. (A) CAD log_2 fold change, tumor versus normal, per cohort with the REML pooled estimate. (B) PTEN–CAD correlations per cohort across all samples (orange) and in primary tumors (black), with pooled estimates. Values in Tables S12 and S15.

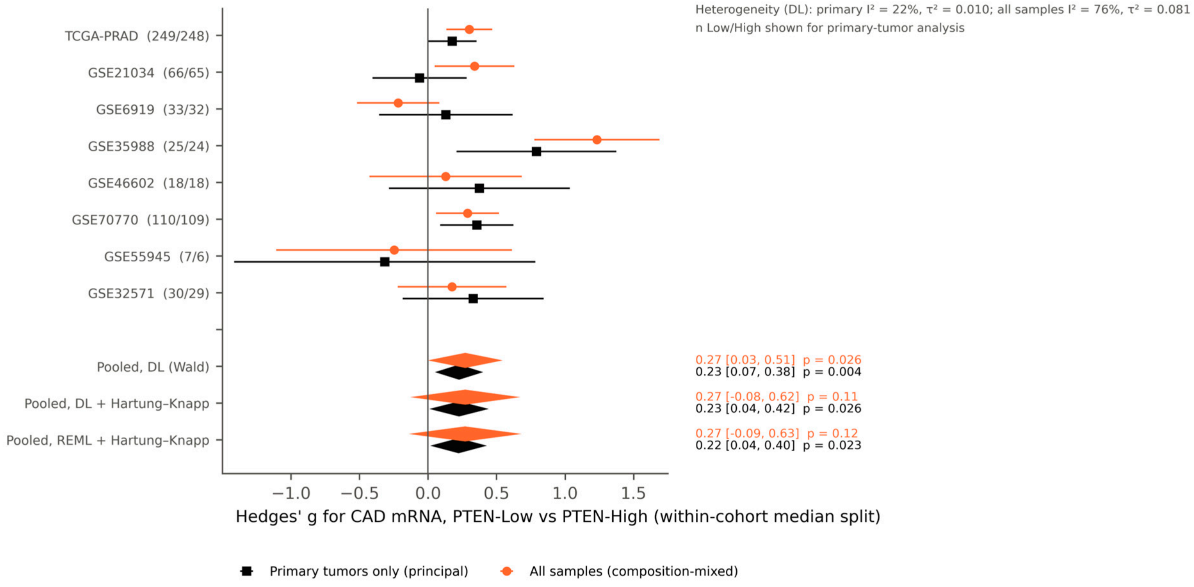

Figure S3. Effect of sample composition: Hedges' g for CAD mRNA, PTEN-Low versus PTEN-High, in primary tumors only (black) and in all samples (orange), with pooled estimates under three estimator/inference combinations. Values in Table 4 and Table S13.

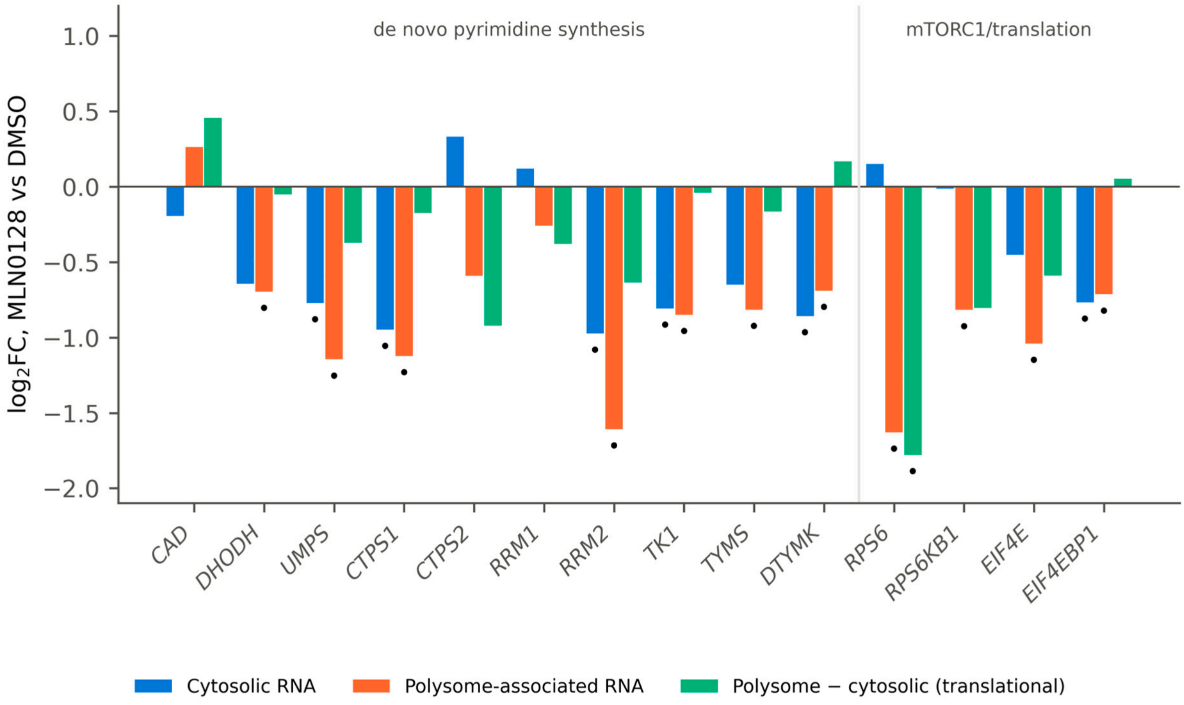

Figure S4. Response of de novo pyrimidine-synthesis and mTORC1/translation transcripts to MLN0128 in MCF7 cells (GSE138417), by RNA fraction: cytosolic (blue), polysome-associated (orange) and their difference (green); dots, FDR < 0.05. Values in Table S18.

Table S1. Gene lists.

Signature	Source	Genes as scored
De novo pyrimidine synthesis	Reactome	CAD, DHODH, UMPS, CTPS2, RRM1, RRM2, TK1, TYMS, DTYMK (CTPS1 absent from HiSeqV2)
mTORC1 signaling	MSigDB HALLMARK_MTORC1_SIGNALING (v2024.1)	All genes except RRM2 (199); nine symbols mapped to HiSeqV2 (EPRS1→EPRS, WARS1→WARS, NHERF1→SLC9A3R1, NIBAN1→FAM129A, AK4→AK3L1, ATP5MC1→ATP5G1, DDX39A→DDX39, ERO1A→ERO1L, SC5D→SC5DL): ABCF2, ACACA, ACLY, ACSL3, ACTR2, ACTR3, ADD3, ADIPOR2, AK4, ALDOA, ARPC5L, ASNS, ATP2A2, ATP5MC1, ATP6V1D, AURKA, BCAT1, BHLHE40, BTG2, BUB1, CACYBP, CALR, CANX, CCNF, CCNG1, CCT6A, CD9, CDC25A, CDKN1A, CFP, COPS5, CORO1A, CTH, CTSC, CXCR4, CYB5B, CYP51A1, DAPP1, DDIT3, DDIT4, DDX39A, DHCR24, DHCR7, DHFR, EBP, EDEM1, EEF1E1, EGLN3, EIF2S2, ELOVL5, ELOVL6, ENO1, EPRS1, ERO1A, ETF1, FADS1, FADS2, FDXR, FGL2, FKBP2, G6PD, GAPDH, GBE1, GCLC, GGA2, GLA, GLRX, GMPS, GOT1, GPI, GSK3B, GSR, GTF2H1, HK2, HMBS, HMGCR, HMGCS1, HPRT1, HSP90B1, HSPA4, HSPA5, HSPA9, HSPD1, HSPE1, IDH1, IDI1, IFI30, IFRD1, IGFBP5, IMMT, INSIG1, ITGB2, LDHA, LDLR, LGMN, LTA4H, M6PR, MAP2K3, MCM2, MCM4, ME1, MLLT11, MTHFD2, MTHFD2L, NAMPT, NFIL3, NFKBIB, NFYC, NHERF1, NIBAN1, NMT1, NUFIP1, NUP205, NUPR1, P4HA1, PDAP1, PDK1, PFKL, PGK1, PGM1, PHGDH, PIK3R3, PITPNB, PLK1, PLOD2, PNO1, PNP, POLR3G, PPA1, PPIA, PPP1R15A, PRDX1, PSAT1, PSMA3, PSMA4, PSMB5, PSMC2, PSMC4, PSMC6, PSMD12, PSMD13, PSMD14, PSME3, PSMG1, PSPH, QDPR, RAB1A, RDH11, RIT1, RPA1, RPN1, RRP9, SC5D, SCD, SDF2L1, SEC11A, SERP1, SERPINH1, SHMT2, SKAP2, SLA, SLC1A4, SLC1A5, SLC2A1, SLC2A3, SLC37A4, SLC6A6, SLC7A11, SLC7A5, SORD, SQLE, SQSTM1, SRD5A1, SSR1, STARD4, STC1, STIP1, SYTL2, TBK1, TCEA1, TES, TFRC, TM7SF2, TMEM97, TOMM40, TPI1, TRIB3, TUBA4A, TUBG1, TXNRD1, UBE2D3, UCHL5, UFM1, UNG, USO1, VLDLR, WARS1, XBP1, YKT6
Proliferation (covariate)	—	MKI67, TOP2A, CCNB1, BUB1, AURKA, PLK1, CDK1, CENPF, UBE2C, BIRC5

Signature	Source	Genes as scored
AR activity (co-variate)	—	KLK3, KLK2, NKX3-1, TMPRSS2, FKBP5, PLPP1, NDRG1, STEAP4, ABCC4, ZBTB16
Alternative mTORC1/SREBP sets	MSigDB c2 v2024.1	BILANGES_RAPAMYCIN_SENSITIVE_GENES (35/37 genes present); BILANGES_RAPAMYCIN_SENSITIVE_VIA_TSC1_AND_TSC2 (64/69 genes present); BILANGES_SERUM_AND_RAPAMYCIN_SENSITIVE_GENES (65/69 genes present); KEGG_MTOR_SIGNALING_PATHWAY (51/52 genes present); KEGG_MEDICUS_REFERENCE_FLCN_MTORC1_SIGNALING_PATHWAY (12/14 genes present); PID_MTOR_4PATHWAY (66/69 genes present); WP_SREBF_AND_MIR33_IN_CHOLESTEROL_AND_LIPID_HOMEOSTASIS (16/18 genes present)

Table S2. Sensitivity of signature results to scoring method (TCGA-PRAD; 159 deleted vs. 326 CN-neutral).

Score	g, deleted vs CN-neutral (95% CI)	Wilcoxon p
mTORC1, GSVA, RRM2 removed (main)	0.543 (0.35 to 0.74)	3.2×10^{-8}
mTORC1, GSVA, all 200 genes	0.550 (0.36 to 0.74)	2.4×10^{-8}
mTORC1, ssGSEA	0.572 (0.38 to 0.77)	2.8×10^{-8}
Pyrimidine, GSVA (main)	0.493 (0.30 to 0.68)	9.1×10^{-7}
Pyrimidine, ssGSEA	0.480 (0.29 to 0.67)	7.1×10^{-7}

Pyrimidine–mTORC1 correlation (n = 485): GSVA r = 0.514; with RRM2 retained r = 0.523; ssGSEA r = 0.481.

Table S3. Pearson correlation of PTEN mRNA with pathway-gene mRNA in TCGA-PRAD.

Module	Gene	r, primary (n = 497)	p	FDR	r, all (n = 550)	p (all)	r, normal (n = 52)	p (normal)
PI3K/AKT	PIK3CA	0.194	1.3×10^{-5}	3.1×10^{-5}	0.237	1.8×10^{-8}	0.625	7.4×10^{-7}
PI3K/AKT	PIK3CB	0.037	0.40	0.46	0.022	0.60	−0.101	0.48
PI3K/AKT	PIK3R1	0.249	1.8×10^{-8}	7.7×10^{-8}	0.319	1.8×10^{-14}	0.568	1.1×10^{-5}
PI3K/AKT	AKT1	−0.010	0.83	0.86	−0.068	0.11	−0.243	0.082
PI3K/AKT	AKT2	−0.120	0.0076	0.011	−0.189	7.7×10^{-6}	−0.214	0.13
PI3K/AKT	AKT3	0.308	2.3×10^{-12}	2.0×10^{-11}	0.326	4.4×10^{-15}	0.370	0.0069
PI3K/AKT	TSC1	0.147	0.0010	0.0020	0.124	0.0035	0.063	0.66
PI3K/AKT	TSC2	−0.243	4.1×10^{-8}	1.5×10^{-7}	−0.243	7.4×10^{-9}	−0.275	0.049
PI3K/AKT	RHEB	0.028	0.53	0.58	0.063	0.14	0.211	0.13
mTORC1/S6K1	MTOR	0.185	3.4×10^{-5}	7.4×10^{-5}	0.140	9.5×10^{-4}	−0.017	0.90
mTORC1/S6K1	RPTOR	0.144	0.0013	0.0024	0.200	2.2×10^{-6}	0.401	0.0032
mTORC1/S6K1	MLST8	−0.325	1.0×10^{-13}	1.3×10^{-12}	−0.382	1.5×10^{-20}	−0.632	4.9×10^{-7}
mTORC1/S6K1	RPS6KB1	0.000	1.00	1.00	−0.016	0.71	0.253	0.070
mTORC1/S6K1	EIF4E	−0.120	0.0075	0.011	−0.113	0.0080	0.039	0.79
mTORC1/S6K1	EIF4EBP1	−0.089	0.046	0.063	−0.144	6.9×10^{-4}	−0.399	0.0034
mTORC1/S6K1	RPS6	0.068	0.13	0.15	0.022	0.60	−0.170	0.23
mTORC1/S6K1	EEF2K	0.452	2.1×10^{-26}	5.5×10^{-25}	0.491	1.1×10^{-34}	0.513	1.0×10^{-4}
Pyrimidine synthesis	CAD	−0.217	1.0×10^{-6}	2.7×10^{-6}	−0.262	4.7×10^{-10}	−0.196	0.16
Pyrimidine synthesis	DHODH	−0.123	0.0062	0.010	−0.165	1.0×10^{-4}	−0.289	0.038
Pyrimidine synthesis	UMPS	−0.257	6.1×10^{-9}	3.2×10^{-8}	−0.300	6.9×10^{-13}	−0.268	0.055
Pyrimidine synthesis	TYMS	−0.088	0.049	0.063	−0.091	0.032	0.051	0.72
Pyrimidine synthesis	CTPS2	0.074	0.10	0.13	0.012	0.79	−0.073	0.61
Pyrimidine synthesis	RRM1	−0.141	0.0016	0.0028	−0.153	3.2×10^{-4}	−0.009	0.95
Pyrimidine synthesis	RRM2	−0.234	1.3×10^{-7}	3.7×10^{-7}	−0.296	1.4×10^{-12}	−0.105	0.46
Pyrimidine synthesis	NME1	−0.239	7.3×10^{-8}	2.4×10^{-7}	−0.317	2.6×10^{-14}	−0.490	2.3×10^{-4}
Pyrimidine synthesis	TK1	−0.259	4.8×10^{-9}	3.1×10^{-8}	−0.320	1.5×10^{-14}	−0.342	0.013

FDR: Benjamini–Hochberg across 26 genes in primary tumors (primary analysis). NME1 is exploratory and not in the signature.

Table S4. Robustness of the TCGA-PRAD associations to the PTEN definition, including the pre-specified mutation-aware definition.

Definition	Outcome	Group (n)	Comparator (n)	Hedges' g (95% CI)	Wilcoxon p
Any deletion vs CN-neutral	CAD	159	326	0.569 (0.38 to 0.76)	7.9×10^{-9}
Deep deletion vs CN-neutral	CAD	95	326	0.668 (0.43 to 0.90)	1.6×10^{-8}
Shallow deletion vs CN-neutral	CAD	64	326	0.417 (0.15 to 0.69)	0.0028
Hybrid: deletion or CN-neutral PTEN mRNA < 30th percentile of CN-neutral	CAD	257	228	0.226 (0.05 to 0.41)	0.011
Hybrid: deletion or CN-neutral PTEN mRNA < 25th percentile of CN-neutral	CAD	241	244	0.264 (0.09 to 0.44)	0.0025
Hybrid: deletion or CN-neutral PTEN mRNA < 20th percentile of CN-neutral	CAD	224	261	0.294 (0.11 to 0.47)	9.2×10^{-4}
Mutation-aware: deletion or non-silent mutation vs CN-neutral and wild-type	CAD mRNA	161	323	0.583 (0.39 to 0.78)	3.2×10^{-9}
Mutation-aware: deletion or non-silent mutation vs CN-neutral and wild-type	mTORC1 score	161	323	0.538 (0.35 to 0.73)	4.2×10^{-8}
Mutation-aware: deletion or non-silent mutation vs CN-neutral and wild-type	Pyrimidine score	161	323	0.497 (0.30 to 0.69)	7.7×10^{-7}

Counts for the mutation-aware definition (MC3 calls; primary tumors in the mutation-calling set):

Item	n
sequenced primary tumours with CN call (gains w/o mutation excluded)	484
PTEN nonsilent mutation (any)	18
mutated & deleted	16
mutated & CN-neutral (reclassified)	2
altered	161
unaltered	323

Mutation-aware models M1–M4 (PTEN-altered coefficient):

Outcome	Model	n	β (95% CI)	p
CAD mRNA	M1	477	0.242 (0.161 to 0.323)	8.8×10^{-9}
CAD mRNA	M2	477	0.206 (0.122 to 0.289)	1.9×10^{-6}
CAD mRNA	M3	477	0.176 (0.095 to 0.257)	2.4×10^{-5}
CAD mRNA	M4	477	0.177 (0.096 to 0.258)	2.2×10^{-5}
mTORC1 score	M1	477	0.123 (0.078 to 0.168)	1.1×10^{-7}
mTORC1 score	M2	477	0.129 (0.082 to 0.176)	9.0×10^{-8}
mTORC1 score	M3	477	0.108 (0.064 to 0.153)	1.8×10^{-6}
mTORC1 score	M4	477	0.114 (0.072 to 0.157)	2.1×10^{-7}
Pyrimidine score	M1	477	0.194 (0.116 to 0.273)	1.4×10^{-6}
Pyrimidine score	M2	477	0.133 (0.054 to 0.212)	0.0010
Pyrimidine score	M3	477	0.066 (0.007 to 0.125)	0.028
Pyrimidine score	M4	477	0.071 (0.012 to 0.130)	0.018

Hybrid rows add CN-neutral tumors with PTEN mRNA below the stated percentile to the deleted group; gains are excluded from every row.

Table S5. CAD locus copy number (GISTIC2) in TCGA-PRAD.

PTEN status	-2	-1	0	1	2
CN-neutral	0	7	308	9	2
deleted	1	14	138	6	0

CAD copy number	n	Mean CAD mRNA
-2	1	9.510
-1	21	9.683
0	446	9.739
1	15	9.969
2	2	9.792

Model	n	PTEN-deletion β (95% CI)	p
CAD ~ PTEN_del	485	0.245 (0.163 to 0.326)	7.1×10^{-9}
CAD ~ PTEN_del + CAD copy number	485	0.256 (0.173 to 0.338)	2.4×10^{-9}

Table S6. Complete coefficients for multivariable models M1–M5 (all classified tumors) and M6 (TCGA 2015 subset).

Outcome	Model	Term	β	95% CI	p	n
CAD mRNA	M1	Intercept	9.6630	9.6161 to 9.7098	$<1\times10^{-300}$	478
CAD mRNA	M1	PTEN deletion	0.2362	0.1548 to 0.3177	2.1×10^{-8}	478
CAD mRNA	M2	Intercept	9.1249	8.7746 to 9.4753	5.8×10^{-195}	478
CAD mRNA	M2	PTEN deletion	0.2000	0.1158 to 0.2841	3.9×10^{-6}	478
CAD mRNA	M2	Age (per year)	0.0060	0.0002 to 0.0117	0.041	478
CAD mRNA	M2	Grade group (per step)	0.0068	−0.0279 to 0.0414	0.70	478
CAD mRNA	M2	pT (per step)	0.0566	0.0016 to 0.1117	0.044	478
CAD mRNA	M3	Intercept	9.3678	9.0237 to 9.7120	1.8×10^{-202}	478
CAD mRNA	M3	PTEN deletion	0.1726	0.0914 to 0.2538	3.5×10^{-5}	478
CAD mRNA	M3	Age (per year)	0.0050	−0.0005 to 0.0105	0.075	478
CAD mRNA	M3	Grade group (per step)	−0.0269	−0.0617 to 0.0078	0.13	478
CAD mRNA	M3	pT (per step)	0.0348	−0.0184 to 0.0881	0.20	478
CAD mRNA	M3	Proliferation score	0.1550	0.1079 to 0.2021	2.5×10^{-10}	478
CAD mRNA	M4	Intercept	9.3538	8.9988 to 9.7088	1.5×10^{-196}	478
CAD mRNA	M4	PTEN deletion	0.1732	0.0919 to 0.2545	3.4×10^{-5}	478
CAD mRNA	M4	Age (per year)	0.0051	−0.0004 to 0.0107	0.070	478
CAD mRNA	M4	Grade group (per step)	−0.0264	−0.0614 to 0.0086	0.14	478
CAD mRNA	M4	pT (per step)	0.0363	−0.0177 to 0.0904	0.19	478
CAD mRNA	M4	Proliferation score	0.1550	0.1079 to 0.2022	2.6×10^{-10}	478
CAD mRNA	M4	AR-activity score	0.0114	−0.0582 to 0.0810	0.75	478
CAD mRNA	M5	Intercept	9.4278	9.0403 to 9.8153	8.8×10^{-168}	409
CAD mRNA	M5	PTEN deletion	0.1639	0.0791 to 0.2486	1.7×10^{-4}	409
CAD mRNA	M5	Age (per year)	0.0046	−0.0014 to 0.0106	0.14	409
CAD mRNA	M5	Grade group (per step)	−0.0383	−0.0765 to −0.0001	0.050	409
CAD mRNA	M5	pT (per step)	0.0446	−0.0145 to 0.1038	0.14	409
CAD mRNA	M5	Proliferation score	0.1478	0.0980 to 0.1976	1.1×10^{-8}	409
CAD mRNA	M5	AR-activity score	−0.0045	−0.0785 to 0.0696	0.91	409
CAD mRNA	M5	pN1 vs pN0	−0.0459	−0.1600 to 0.0681	0.43	409
mTORC1 score	M1	Intercept	−0.0639	−0.0897 to −0.0380	1.6×10^{-6}	478
mTORC1 score	M1	PTEN deletion	0.1244	0.0794 to 0.1694	8.7×10^{-8}	478
mTORC1 score	M2	Intercept	0.1032	−0.0922 to 0.2987	0.30	478
mTORC1 score	M2	PTEN deletion	0.1303	0.0834 to 0.1773	7.9×10^{-8}	478
mTORC1 score	M2	Age (per year)	−0.0023	−0.0055 to 0.0009	0.16	478
mTORC1 score	M2	Grade group (per step)	0.0035	−0.0159 to 0.0228	0.72	478
mTORC1 score	M2	pT (per step)	−0.0142	−0.0449 to 0.0165	0.36	478
mTORC1 score	M3	Intercept	0.2723	0.0851 to 0.4595	0.0045	478
mTORC1 score	M3	PTEN deletion	0.1113	0.0671 to 0.1554	1.0×10^{-6}	478
mTORC1 score	M3	Age (per year)	−0.0029	−0.0059 to 0.0000	0.054	478
mTORC1 score	M3	Grade group (per step)	−0.0200	−0.0389 to −0.0011	0.038	478
mTORC1 score	M3	pT (per step)	−0.0294	−0.0583 to −0.0004	0.047	478

Outcome	Model	Term	β	95% CI	p	n
mTORC1 score	M3	Proliferation score	0.1079	0.0823 to 0.1335	$1.3×10^{−15}$	478
mTORC1 score	M4	Intercept	0.1367	−0.0496 to 0.3230	0.15	478
mTORC1 score	M4	PTEN deletion	0.1172	0.0745 to 0.1599	$1.1×10^{−7}$	478
mTORC1 score	M4	Age (per year)	−0.0017	−0.0046 to 0.0012	0.24	478
mTORC1 score	M4	Grade group (per step)	−0.0148	−0.0332 to 0.0035	0.11	478
mTORC1 score	M4	pT (per step)	−0.0150	−0.0434 to 0.0134	0.30	478
mTORC1 score	M4	Proliferation score	0.1083	0.0835 to 0.1330	$1.2×10^{−16}$	478
mTORC1 score	M4	AR-activity score	0.1102	0.0737 to 0.1468	$5.9×10^{−9}$	478
mTORC1 score	M5	Intercept	0.1434	−0.0609 to 0.3477	0.17	409
mTORC1 score	M5	PTEN deletion	0.1143	0.0697 to 0.1590	$7.4×10^{−7}$	409
mTORC1 score	M5	Age (per year)	−0.0022	−0.0054 to 0.0010	0.17	409
mTORC1 score	M5	Grade group (per step)	−0.0119	−0.0321 to 0.0082	0.24	409
mTORC1 score	M5	pT (per step)	−0.0125	−0.0437 to 0.0187	0.43	409
mTORC1 score	M5	Proliferation score	0.1089	0.0826 to 0.1351	$4.5×10^{−15}$	409
mTORC1 score	M5	AR-activity score	0.1063	0.0672 to 0.1453	$1.5×10^{−7}$	409
mTORC1 score	M5	pN1 vs pN0	0.0042	−0.0560 to 0.0643	0.89	409
Pyrimidine score	M1	Intercept	−0.1080	−0.1532 to −0.0628	$3.4×10^{−6}$	478
Pyrimidine score	M1	PTEN deletion	0.1931	0.1145 to 0.2717	$1.9×10^{−6}$	478
Pyrimidine score	M2	Intercept	−0.6710	−1.0023 to −0.3398	$8.0×10^{−5}$	478
Pyrimidine score	M2	PTEN deletion	0.1305	0.0509 to 0.2100	0.0014	478
Pyrimidine score	M2	Age (per year)	0.0046	−0.0008 to 0.0100	0.094	478
Pyrimidine score	M2	Grade group (per step)	0.0485	0.0157 to 0.0812	0.0038	478
Pyrimidine score	M2	pT (per step)	0.0500	−0.0020 to 0.1021	0.060	478
Pyrimidine score	M3	Intercept	−0.1302	−0.3816 to 0.1213	0.31	478
Pyrimidine score	M3	PTEN deletion	0.0695	0.0102 to 0.1288	0.022	478
Pyrimidine score	M3	Age (per year)	0.0025	−0.0015 to 0.0065	0.22	478
Pyrimidine score	M3	Grade group (per step)	−0.0266	−0.0520 to −0.0012	0.040	478
Pyrimidine score	M3	pT (per step)	0.0016	−0.0373 to 0.0404	0.94	478
Pyrimidine score	M3	Proliferation score	0.3452	0.3107 to 0.3796	$1.5×10^{−63}$	478
Pyrimidine score	M4	Intercept	−0.2387	−0.4949 to 0.0175	0.068	478
Pyrimidine score	M4	PTEN deletion	0.0743	0.0156 to 0.1330	0.013	478
Pyrimidine score	M4	Age (per year)	0.0035	−0.0005 to 0.0075	0.090	478
Pyrimidine score	M4	Grade group (per step)	−0.0225	−0.0477 to 0.0028	0.081	478
Pyrimidine score	M4	pT (per step)	0.0131	−0.0259 to 0.0521	0.51	478
Pyrimidine score	M4	Proliferation score	0.3455	0.3114 to 0.3795	$1.2×10^{−64}$	478
Pyrimidine score	M4	AR-activity score	0.0883	0.0380 to 0.1385	$6.1×10^{−4}$	478
Pyrimidine score	M5	Intercept	−0.2589	−0.5429 to 0.0250	0.074	409
Pyrimidine score	M5	PTEN deletion	0.0714	0.0093 to 0.1335	0.024	409
Pyrimidine score	M5	Age (per year)	0.0028	−0.0016 to 0.0072	0.21	409
Pyrimidine score	M5	Grade group (per step)	−0.0111	−0.0392 to 0.0169	0.44	409
Pyrimidine score	M5	pT (per step)	0.0229	−0.0205 to 0.0662	0.30	409
Pyrimidine score	M5	Proliferation score	0.3505	0.3141 to 0.3870	$2.1×10^{−57}$	409
Pyrimidine score	M5	AR-activity score	0.1020	0.0477 to 0.1562	$2.5×10^{−4}$	409
Pyrimidine score	M5	pN1 vs pN0	−0.0555	−0.1391 to 0.0281	0.19	409
CAD mRNA	M6	Intercept	8.9526	8.5377 to 9.3674	$2.2×10^{−116}$	276
CAD mRNA	M6	PTEN deletion	−0.0021	−0.0950 to 0.0908	0.96	276
CAD mRNA	M6	Age (per year)	0.0094	0.0034 to 0.0154	0.0023	276
CAD mRNA	M6	Grade group (per step)	0.0078	−0.0322 to 0.0477	0.70	276
CAD mRNA	M6	pT (per step)	0.0478	−0.0118 to 0.1075	0.12	276
CAD mRNA	M6	Proliferation score	0.1653	0.1068 to 0.2238	$6.7×10^{−8}$	276
CAD mRNA	M6	AR-activity score	0.0282	−0.0810 to 0.1374	0.61	276
CAD mRNA	M6	ABSOLUTE purity	0.2496	0.0286 to 0.4707	0.027	276
CAD mRNA	M6	TP53 altered	−0.0086	−0.1604 to 0.1433	0.91	276
CAD mRNA	M6	RB1 altered	0.1468	−0.2385 to 0.5321	0.45	276

Outcome	Model	Term	β	95% CI	p	n
CAD mRNA	M6	ERG fusion	0.2502	0.1633 to 0.3371	$3.9×10^{-8}$	276
mTORC1 score	M6	Intercept	0.1126	−0.1474 to 0.3725	0.39	276
mTORC1 score	M6	PTEN deletion	0.1446	0.0863 to 0.2028	$1.8×10^{-6}$	276
mTORC1 score	M6	Age (per year)	−0.0019	−0.0057 to 0.0018	0.31	276
mTORC1 score	M6	Grade group (per step)	−0.0265	−0.0515 to −0.0014	0.038	276
mTORC1 score	M6	pT (per step)	−0.0104	−0.0478 to 0.0269	0.58	276
mTORC1 score	M6	Proliferation score	0.0786	0.0419 to 0.1152	$3.4×10^{-5}$	276
mTORC1 score	M6	AR-activity score	0.1290	0.0605 to 0.1974	$2.6×10^{-4}$	276
mTORC1 score	M6	ABSOLUTE purity	0.2057	0.0672 to 0.3442	0.0038	276
mTORC1 score	M6	TP53 altered	0.0209	−0.0742 to 0.1161	0.67	276
mTORC1 score	M6	RB1 altered	−0.0996	−0.3410 to 0.1419	0.42	276
mTORC1 score	M6	ERG fusion	−0.0820	−0.1365 to −0.0276	0.0033	276
Pyrimidine score	M6	Intercept	−0.4784	−0.8180 to −0.1389	0.0059	276
Pyrimidine score	M6	PTEN deletion	0.0768	0.0008 to 0.1529	0.048	276
Pyrimidine score	M6	Age (per year)	0.0032	−0.0017 to 0.0082	0.19	276
Pyrimidine score	M6	Grade group (per step)	−0.0224	−0.0551 to 0.0102	0.18	276
Pyrimidine score	M6	pT (per step)	0.0351	−0.0138 to 0.0839	0.16	276
Pyrimidine score	M6	Proliferation score	0.3565	0.3086 to 0.4044	$1.5×10^{-35}$	276
Pyrimidine score	M6	AR-activity score	0.1667	0.0773 to 0.2561	$2.9×10^{-4}$	276
Pyrimidine score	M6	ABSOLUTE purity	0.1903	0.0094 to 0.3712	0.039	276
Pyrimidine score	M6	TP53 altered	0.0399	−0.0844 to 0.1641	0.53	276
Pyrimidine score	M6	RB1 altered	0.0325	−0.2829 to 0.3479	0.84	276
Pyrimidine score	M6	ERG fusion	0.0250	−0.0461 to 0.0961	0.49	276

M5 adds pathologic N stage to M4 (n = 409). M6 adds purity, TP53, RB1, ERG fusion and sequencing batch (batch coefficients, one per batch, are in Supplementary Data S1).

Table S7. Exploratory: all RPPA antibodies, PTEN-deleted versus CN-neutral (193 antibodies with data).

Antibody	Hedges' g (95% CI)	Wilcoxon p	FDR
PKC-delta_pS664	−0.534 (−0.76 to −0.31)	$3.6×10^{-7}$	$6.9×10^{-5}$
INPP4B	−0.537 (−0.76 to −0.31)	$1.1×10^{-6}$	$1.0×10^{-4}$
STAT5-alpha	0.528 (0.30 to 0.75)	$1.6×10^{-6}$	$1.0×10^{-4}$
53BP1	0.528 (0.30 to 0.75)	$2.2×10^{-6}$	$1.1×10^{-4}$
Myosin-IIa-pS1943	0.470 (0.24 to 0.70)	$1.7×10^{-5}$	$6.6×10^{-4}$
GAB2	0.490 (0.26 to 0.72)	$2.3×10^{-5}$	$7.5×10^{-4}$
PKC-alpha	−0.446 (−0.67 to −0.22)	$7.4×10^{-5}$	0.0020
PKC-alpha_pS657	−0.414 (−0.64 to −0.19)	$8.6×10^{-5}$	0.0021
PTEN	−0.456 (−0.68 to −0.23)	$9.6×10^{-5}$	0.0021
Dvl3	0.414 (0.19 to 0.64)	$1.5×10^{-4}$	0.0030
PREX1	0.337 (0.11 to 0.56)	$2.1×10^{-4}$	0.0031
CD49b	−0.459 (−0.68 to −0.23)	$2.1×10^{-4}$	0.0031
AMPK_pT172	0.380 (0.16 to 0.60)	$2.1×10^{-4}$	0.0031
Syk	0.340 (0.12 to 0.56)	$4.0×10^{-4}$	0.0055
DJ-1	−0.398 (−0.62 to −0.17)	$4.4×10^{-4}$	0.0056
DIRAS3	−0.398 (−0.62 to −0.17)	$4.7×10^{-4}$	0.0057
Shc_pY317	−0.427 (−0.65 to −0.20)	$5.9×10^{-4}$	0.0064
RBM15	0.373 (0.15 to 0.60)	$6.0×10^{-4}$	0.0064
P-Cadherin	−0.405 (−0.63 to −0.18)	$9.0×10^{-4}$	0.0092
TFRC	0.350 (0.13 to 0.57)	0.0010	0.0099
p62-LCK-ligand	0.369 (0.14 to 0.59)	0.0011	0.0099
Ku80	0.345 (0.12 to 0.57)	0.0012	0.0099
SCD1	−0.269 (−0.49 to −0.05)	0.0012	0.0099
Smad4	−0.359 (−0.58 to −0.13)	0.0013	0.010

Antibody	Hedges’ g (95% CI)	Wilcoxon p	FDR
ACVRL1	−0.352 (−0.58 to −0.13)	0.0013	0.010
PKC-pan_BetaII_pS660	−0.354 (−0.58 to −0.13)	0.0017	0.012
Caveolin-1	−0.287 (−0.51 to −0.06)	0.0017	0.012
IGFBP2	−0.335 (−0.56 to −0.11)	0.0024	0.017
eIF4G	0.358 (0.13 to 0.58)	0.0026	0.017
PRDX1	−0.356 (−0.58 to −0.13)	0.0029	0.019
PDCD4	−0.369 (−0.59 to −0.14)	0.0033	0.020
N-Ras	−0.343 (−0.57 to −0.12)	0.0039	0.024
Akt_pS473	0.324 (0.10 to 0.55)	0.0041	0.024
EGFR_pY1173	−0.312 (−0.54 to −0.09)	0.0042	0.024
BRCA2	−0.322 (−0.55 to −0.10)	0.0046	0.026
LKB1	−0.310 (−0.53 to −0.09)	0.0053	0.028
PAI-1	0.263 (0.04 to 0.49)	0.0055	0.028
VHL	0.303 (0.08 to 0.53)	0.0063	0.032
C-Raf_pS338	−0.304 (−0.53 to −0.08)	0.0064	0.032
14-3-3_epsilon	−0.295 (−0.52 to −0.07)	0.0092	0.044
ATM	0.302 (0.08 to 0.53)	0.011	0.051
p90RSK_pT359_S363	−0.328 (−0.55 to −0.10)	0.012	0.051
PDK1_pS241	−0.261 (−0.48 to −0.04)	0.012	0.051
ASNS	0.335 (0.11 to 0.56)	0.012	0.051
Chk2_pT68	−0.227 (−0.45 to −0.00)	0.012	0.051
PR	−0.276 (−0.50 to −0.05)	0.012	0.051
Heregulin	−0.246 (−0.47 to −0.02)	0.012	0.051
MEK1_pS217_S221	−0.292 (−0.52 to −0.07)	0.015	0.059
Mre11	−0.278 (−0.50 to −0.05)	0.016	0.061
AR	−0.220 (−0.44 to 0.00)	0.016	0.061
Src	−0.294 (−0.52 to −0.07)	0.016	0.061
p27	−0.264 (−0.49 to −0.04)	0.017	0.061
p38_MAPK	0.168 (−0.06 to 0.39)	0.017	0.061
ER-alpha	0.295 (0.07 to 0.52)	0.017	0.061
Paxillin	0.251 (0.03 to 0.47)	0.017	0.061
p53	−0.273 (−0.50 to −0.05)	0.018	0.062
CD31	−0.277 (−0.50 to −0.05)	0.018	0.062
Bad_pS112	0.193 (−0.03 to 0.42)	0.019	0.064
MSH6	0.240 (0.02 to 0.46)	0.023	0.077
TAZ	0.106 (−0.12 to 0.33)	0.024	0.078
c-Kit	−0.323 (−0.55 to −0.10)	0.027	0.084
HER3_pY1298	−0.233 (−0.46 to −0.01)	0.030	0.094
Src_pY416	−0.238 (−0.46 to −0.01)	0.031	0.094
GSK3-alpha-beta	0.233 (0.01 to 0.46)	0.032	0.097
Rictor_pT1135	−0.217 (−0.44 to 0.01)	0.033	0.098
EGFR_pY1068	−0.273 (−0.50 to −0.05)	0.034	0.099
p70S6K_pT389	−0.261 (−0.49 to −0.04)	0.036	0.10
Rab11	−0.246 (−0.47 to −0.02)	0.037	0.10
N-Cadherin	−0.226 (−0.45 to −0.00)	0.038	0.10
Beclin	−0.253 (−0.48 to −0.03)	0.038	0.10
Transglutaminase	0.237 (0.01 to 0.46)	0.039	0.10
Akt_pT308	0.251 (0.03 to 0.48)	0.039	0.10
Bcl-2	−0.277 (−0.50 to −0.05)	0.041	0.11
FOXO3a_pS318_S321	−0.198 (−0.42 to 0.03)	0.042	0.11
c-Myc	−0.238 (−0.46 to −0.01)	0.044	0.11
ERK2	−0.182 (−0.41 to 0.04)	0.046	0.12
Bak	−0.236 (−0.46 to −0.01)	0.049	0.12
FoxM1	−0.200 (−0.42 to 0.02)	0.052	0.13

Antibody	Hedges’ g (95% CI)	Wilcoxon p	FDR
Bax	0.196 (−0.03 to 0.42)	0.054	0.13
Tuberin	0.282 (0.06 to 0.51)	0.058	0.14
NF2	−0.184 (−0.41 to 0.04)	0.059	0.14
Cyclin_E1	−0.264 (−0.49 to −0.04)	0.062	0.15
Stathmin	−0.194 (−0.42 to 0.03)	0.068	0.16
Tuberin_pT1462	−0.195 (−0.42 to 0.03)	0.070	0.16
c-Met_pY1235	−0.179 (−0.40 to 0.04)	0.074	0.17
Bid	−0.171 (−0.39 to 0.05)	0.077	0.17
PEA15	−0.095 (−0.32 to 0.13)	0.078	0.17
FOXO3a	−0.270 (−0.49 to −0.05)	0.082	0.18
AMPK_alpha	−0.190 (−0.41 to 0.03)	0.084	0.18
GATA3	−0.204 (−0.43 to 0.02)	0.085	0.18
YB-1_pS102	−0.259 (−0.48 to −0.04)	0.091	0.19
eEF2	0.146 (−0.08 to 0.37)	0.093	0.20
alpha-Catenin	−0.257 (−0.48 to −0.03)	0.099	0.20
p70S6K	0.152 (−0.07 to 0.38)	0.11	0.23
Akt	−0.133 (−0.36 to 0.09)	0.12	0.25
Annexin_VII	0.077 (−0.15 to 0.30)	0.12	0.25
VEGFR2	−0.156 (−0.38 to 0.07)	0.12	0.25
ACC1	0.182 (−0.04 to 0.41)	0.14	0.27
JNK_pT183_Y185	−0.152 (−0.38 to 0.07)	0.14	0.27
NDRG1_pT346	0.262 (0.04 to 0.49)	0.14	0.27
p21	−0.182 (−0.41 to 0.04)	0.15	0.30
PDK1	−0.181 (−0.40 to 0.04)	0.18	0.33
XRCC1	0.154 (−0.07 to 0.38)	0.18	0.33
c-Jun_pS73	−0.173 (−0.40 to 0.05)	0.18	0.33
ACC_pS79	0.157 (−0.07 to 0.38)	0.21	0.39
beta-Catenin	0.157 (−0.07 to 0.38)	0.21	0.39
CD20	−0.090 (−0.31 to 0.13)	0.21	0.39
cIAP	0.103 (−0.12 to 0.33)	0.22	0.39
TTF1	−0.122 (−0.35 to 0.10)	0.22	0.39
eIF4E	−0.102 (−0.33 to 0.12)	0.23	0.40
Rictor	−0.107 (−0.33 to 0.12)	0.23	0.41
Collagen_VI	−0.133 (−0.36 to 0.09)	0.25	0.43
Bcl-xL	−0.158 (−0.38 to 0.07)	0.25	0.43
Bim	0.099 (−0.12 to 0.32)	0.26	0.44
B-Raf	0.081 (−0.14 to 0.30)	0.26	0.44
4E-BP1_pT37T46	0.096 (−0.13 to 0.32)	0.27	0.44
XBP1	−0.140 (−0.36 to 0.08)	0.27	0.44
Annexin-1	0.151 (−0.07 to 0.37)	0.27	0.44
MIG-6	−0.117 (−0.34 to 0.11)	0.28	0.44
A-Raf_pS299	−0.055 (−0.28 to 0.17)	0.28	0.44
Chk1	−0.064 (−0.29 to 0.16)	0.28	0.45
14-3-3_beta	−0.106 (−0.33 to 0.12)	0.29	0.46
HSP70	−0.159 (−0.38 to 0.06)	0.29	0.46
4E-BP1_pT70	0.164 (−0.06 to 0.39)	0.30	0.46
PI3K-p85	−0.085 (−0.31 to 0.14)	0.31	0.49
PCNA	−0.077 (−0.30 to 0.15)	0.33	0.50
ADAR1	−0.095 (−0.32 to 0.13)	0.36	0.54
Src_pY527	−0.093 (−0.32 to 0.13)	0.36	0.54
Rad51	−0.081 (−0.30 to 0.14)	0.36	0.54
4E-BP1_pS65	−0.091 (−0.31 to 0.13)	0.37	0.54
Caspase-7_cleavedD198	−0.048 (−0.27 to 0.17)	0.38	0.56
YAP_pS127	−0.045 (−0.27 to 0.18)	0.39	0.57

Antibody	Hedges' g (95% CI)	Wilcoxon p	FDR
14-3-3_zeta	0.130 (−0.09 to 0.35)	0.39	0.57
TSC1	0.114 (−0.11 to 0.34)	0.40	0.57
YAP	−0.111 (−0.33 to 0.11)	0.42	0.59
Cyclin_E2	−0.046 (−0.27 to 0.18)	0.42	0.59
GSK3-alpha-beta_pS21_S9	−0.057 (−0.28 to 0.17)	0.43	0.60
Rb_pS807_S811	0.085 (−0.14 to 0.31)	0.43	0.61
Smac	0.078 (−0.15 to 0.30)	0.46	0.64
IRS1	−0.086 (−0.31 to 0.14)	0.50	0.68
EGFR	0.092 (−0.13 to 0.32)	0.51	0.69
p27_pT157	−0.051 (−0.27 to 0.17)	0.51	0.69
PEA15_pS116	−0.042 (−0.26 to 0.18)	0.52	0.69
Chk2	−0.107 (−0.33 to 0.12)	0.52	0.69
p38_pT180_Y182	−0.040 (−0.26 to 0.18)	0.53	0.70
STAT3_pY705	−0.003 (−0.23 to 0.22)	0.53	0.70
Bap1-c-4	0.027 (−0.20 to 0.25)	0.54	0.71
Fibronectin	−0.027 (−0.25 to 0.20)	0.57	0.74
C-Raf	0.114 (−0.11 to 0.34)	0.61	0.77
NF-kB-p65_pS536	0.052 (−0.17 to 0.27)	0.61	0.77
Cyclin_D1	−0.016 (−0.24 to 0.21)	0.61	0.77
CDK1	−0.032 (−0.26 to 0.19)	0.61	0.77
Rad50	−0.131 (−0.35 to 0.09)	0.62	0.78
Lck	0.007 (−0.22 to 0.23)	0.62	0.78
p27_pT198	−0.144 (−0.37 to 0.08)	0.63	0.78
4E-BP1	0.027 (−0.20 to 0.25)	0.64	0.79
MEK1	−0.032 (−0.26 to 0.19)	0.65	0.79
GSK3_pS9	−0.011 (−0.23 to 0.21)	0.65	0.79
eEF2K	−0.058 (−0.28 to 0.17)	0.66	0.80
Rab25	−0.032 (−0.25 to 0.19)	0.66	0.80
TIGAR	0.076 (−0.15 to 0.30)	0.68	0.81
SF2	−0.007 (−0.23 to 0.22)	0.68	0.81
Smad3	−0.034 (−0.26 to 0.19)	0.69	0.81
G6PD	0.038 (−0.19 to 0.26)	0.69	0.81
ER-alpha_pS118	0.033 (−0.19 to 0.26)	0.69	0.81
S6_pS240_S244	0.033 (−0.19 to 0.26)	0.70	0.81
JNK2	0.020 (−0.20 to 0.24)	0.71	0.82
MSH2	−0.160 (−0.38 to 0.06)	0.74	0.85
MAPK_pT202_Y204	−0.050 (−0.27 to 0.17)	0.75	0.85
mTOR	−0.006 (−0.23 to 0.22)	0.77	0.87
S6	−0.009 (−0.23 to 0.21)	0.77	0.87
mTOR_pS2448	0.034 (−0.19 to 0.26)	0.78	0.87
Smad1	0.061 (−0.16 to 0.28)	0.80	0.89
Acetyl-a-Tubulin-Lys40	0.043 (−0.18 to 0.27)	0.80	0.89
p90RSK	0.041 (−0.18 to 0.26)	0.81	0.89
HER3	0.043 (−0.18 to 0.27)	0.82	0.89
Raptor	−0.005 (−0.23 to 0.22)	0.82	0.89
Cyclin_B1	−0.013 (−0.24 to 0.21)	0.85	0.92
ERCC1	0.013 (−0.21 to 0.24)	0.87	0.93
E-Cadherin	−0.002 (−0.22 to 0.22)	0.87	0.93
FASN	0.007 (−0.22 to 0.23)	0.88	0.94
Notch1	−0.022 (−0.24 to 0.20)	0.89	0.94
ETS-1	−0.012 (−0.23 to 0.21)	0.89	0.94
PRAS40_pT246	0.022 (−0.20 to 0.24)	0.91	0.95
YB-1	−0.001 (−0.22 to 0.22)	0.94	0.97
MYH11	0.025 (−0.20 to 0.25)	0.94	0.97

Antibody	Hedges' g (95% CI)	Wilcoxon p	FDR
HER2	−0.014 (−0.24 to 0.21)	0.94	0.97
PI3K-p110-alpha	0.041 (−0.18 to 0.26)	0.95	0.97
HER2_pY1248	0.031 (−0.19 to 0.25)	0.95	0.97
GAPDH	−0.032 (−0.25 to 0.19)	0.96	0.97
Chk1_pS345	0.016 (−0.21 to 0.24)	0.98	0.99
Claudin-7	0.007 (−0.22 to 0.23)	0.99	0.99
S6_pS235_S236	−0.001 (−0.22 to 0.22)	0.99	0.99

FDR is Benjamini–Hochberg across all antibodies. Results are exploratory.

Table S8. Pre-specified protein-level analysis (TCGA-PRAD RPPA; 118 PTEN-deleted, 224 CN-neutral).

Antibody / endpoint	Mean, deleted	Mean, CN-neutral	Hedges' g (95% CI)	Wilcoxon p	FDR (family)
mTORC1 phospho-output score (6 antibodies)	−0.006	0.003	−0.017 (−0.24 to 0.21)	0.94	—
PTEN	0.105	0.361	−0.456 (−0.68 to −0.23)	9.6×10^{-5}	9.6×10^{-4}
Akt_pS473	−0.044	−0.284	0.324 (0.10 to 0.55)	0.0041	0.020
Akt_pT308	0.416	0.279	0.251 (0.03 to 0.48)	0.039	0.097
mTOR_pS2448	−0.410	−0.418	0.034 (−0.19 to 0.26)	0.78	0.87
p70S6K_pT389	−1.054	−0.945	−0.261 (−0.49 to −0.04)	0.036	0.097
S6_pS235_S236	−1.068	−1.068	−0.001 (−0.22 to 0.22)	0.99	0.99
S6_pS240_S244	−0.926	−0.949	0.033 (−0.19 to 0.26)	0.70	0.87
4E-BP1_pS65	0.215	0.240	−0.091 (−0.31 to 0.13)	0.37	0.52
4E-BP1_pT37T46	0.460	0.391	0.096 (−0.13 to 0.32)	0.27	0.49
4E-BP1_pT70	−0.301	−0.344	0.164 (−0.06 to 0.39)	0.30	0.49

Models for PTEN protein and the phospho-output score (PTEN-deletion coefficient):

Outcome	Model	n	β (95% CI)	p
composite	M1	337	−0.001 (−0.132 to 0.130)	0.99
composite	M2	337	0.050 (−0.083 to 0.183)	0.46
composite	M3	337	0.045 (−0.089 to 0.180)	0.51
composite	M4	337	0.045 (−0.089 to 0.180)	0.51
PTEN protein	M1	337	−0.261 (−0.387 to −0.135)	6.0×10^{-5}
PTEN protein	M2	337	−0.230 (−0.361 to −0.099)	6.3×10^{-4}
PTEN protein	M3	337	−0.239 (−0.372 to −0.107)	4.3×10^{-4}
PTEN protein	M4	337	−0.238 (−0.370 to −0.106)	4.3×10^{-4}

Correlations:

Pair	n	r (95% CI)	p
PTEN mRNA vs PTEN protein	342	0.350 (0.25 to 0.44)	2.6×10^{-11}
mTORC1 GSVA score vs mTORC1 phospho-output score	342	0.024 (−0.08 to 0.13)	0.65
CAD mRNA vs mTORC1 phospho-output score	342	0.082 (−0.02 to 0.19)	0.13
Pyrimidine score vs mTORC1 phospho-output score (exploratory, not pre-specified)	342	0.052 (−0.05 to 0.16)	0.33
PTEN protein vs mTORC1 phospho-output score (exploratory, not pre-specified)	342	−0.250 (−0.35 to −0.15)	2.7×10^{-6}

The phospho-output score (pre-specified primary protein endpoint) is the mean Z-score of the six mTORC1-output antibodies; FDR is within the 10-antibody pre-specified family. CAD is not on the RPPA panel.

Table S9. Alternative mTORC1- and SREBP-related gene sets (MSigDB c2), PTEN-deleted (n = 159) versus CN-neutral (n = 326).

Gene set	Genes present	Hedges' g (95% CI)	Wilcoxon p	FDR
BILANGES_RAPAMYCIN_SENSITIVE_GENES	35/37	−0.073 (−0.26 to 0.12)	0.45	0.52
BILANGES_RAPAMYCIN_SENSITIVE_VIA_TSC1_AND_TSC2	64/69	0.066 (−0.12 to 0.26)	0.40	0.52
BILANGES_SERUM_AND_RAPAMYCIN_SENSITIVE_GENES	65/69	−0.223 (−0.41 to −0.03)	0.015	0.10
KEGG_MTOR_SIGNALING_PATHWAY	51/52	−0.134 (−0.32 to 0.06)	0.21	0.38

Gene set	Genes present	Hedges' g (95% CI)	Wilcoxon p	FDR
KEGG_MEDICUS_REFERENCE_FLCN_MTORC1_SIGNALING_PATHWAY	12/14	0.001 (−0.19 to 0.19)	0.96	0.96
PID_MTOR_4PATHWAY	66/69	0.168 (−0.02 to 0.36)	0.075	0.26
WP_SREBF_AND_MIR33_IN_CHOLESTEROL_AND_LIPID_HOMEOSTASIS	16/18	−0.132 (−0.32 to 0.06)	0.22	0.38

KEGG and PID sets list pathway components rather than transcriptional outputs.

Table S10. Post hoc ERG analyses.

(a) PTEN deletion and ERG fusion (TCGA 2015 subset): PTEN-deleted 74/110 ERG-fusion-positive versus CN-neutral 78/217; odds ratio 3.65 (95% CI 2.20–6.14), Fisher p = 9.9×10^{-8}. ERG mRNA as a proxy for ERG fusion (n = 333, 152 fusion-positive): AUC = 0.997; two-cluster ERG-high agreement with fusion status = 98.2%.

(b) Decomposition (PTEN-deletion coefficient; 2015 subset with purity data unless stated).

Outcome	Model	n	β (95% CI)	p
CAD mRNA	M4 (purity-available subset)	276	0.127 (0.032 to 0.222)	0.0090
CAD mRNA	M4 + ERG	276	0.036 (−0.059 to 0.131)	0.46
CAD mRNA	M4 + purity	276	0.126 (0.031 to 0.220)	0.0094
CAD mRNA	M4 + TP53 + RB1	276	0.121 (0.025 to 0.217)	0.014
CAD mRNA	M4 + batch	276	0.085 (−0.009 to 0.179)	0.076
CAD mRNA	M4 + ERG + purity	276	0.036 (−0.058 to 0.131)	0.45
CAD mRNA	M4 + ERG (all 2015 tumours)	322	0.041 (−0.047 to 0.129)	0.36
CAD mRNA	M4 within ERG-fusion tumours	151	0.027 (−0.088 to 0.143)	0.64
CAD mRNA	M4 within ERG-negative tumours	171	0.038 (−0.097 to 0.174)	0.58
CAD mRNA	M4 + any ETS fusion (ERG/ETV1/ETV4/FLI1)	322	0.017 (−0.068 to 0.102)	0.70
mTORC1 score	M4 (purity-available subset)	276	0.133 (0.077 to 0.190)	5.7×10^{-6}
mTORC1 score	M4 + ERG	276	0.158 (0.098 to 0.217)	3.4×10^{-7}
mTORC1 score	M4 + purity	276	0.133 (0.076 to 0.189)	6.0×10^{-6}
mTORC1 score	M4 + TP53 + RB1	276	0.135 (0.078 to 0.193)	5.2×10^{-6}
mTORC1 score	M4 + batch	276	0.123 (0.066 to 0.180)	2.7×10^{-5}
mTORC1 score	M4 + ERG + purity	276	0.158 (0.099 to 0.217)	3.0×10^{-7}
mTORC1 score	M4 + ERG (all 2015 tumours)	322	0.162 (0.106 to 0.217)	2.4×10^{-8}
mTORC1 score	M4 within ERG-fusion tumours	151	0.176 (0.106 to 0.246)	2.0×10^{-6}
mTORC1 score	M4 within ERG-negative tumours	171	0.150 (0.060 to 0.240)	0.0013
mTORC1 score	M4 + any ETS fusion (ERG/ETV1/ETV4/FLI1)	322	0.165 (0.110 to 0.221)	1.3×10^{-8}
Pyrimidine score	M4 (purity-available subset)	276	0.070 (−0.002 to 0.141)	0.055
Pyrimidine score	M4 + ERG	276	0.063 (−0.012 to 0.139)	0.100
Pyrimidine score	M4 + purity	276	0.068 (−0.002 to 0.139)	0.057
Pyrimidine score	M4 + TP53 + RB1	276	0.066 (−0.006 to 0.139)	0.071
Pyrimidine score	M4 + batch	276	0.093 (0.020 to 0.165)	0.012
Pyrimidine score	M4 + ERG + purity	276	0.064 (−0.011 to 0.139)	0.093
Pyrimidine score	M4 + ERG (all 2015 tumours)	322	0.062 (−0.007 to 0.131)	0.080
Pyrimidine score	M4 within ERG-fusion tumours	151	0.098 (0.003 to 0.193)	0.043
Pyrimidine score	M4 within ERG-negative tumours	171	0.009 (−0.096 to 0.114)	0.87
Pyrimidine score	M4 + any ETS fusion (ERG/ETV1/ETV4/FLI1)	322	0.066 (−0.004 to 0.136)	0.063

(c) All classified tumors, M4 with and without ERG mRNA.

Outcome	Model	n	β (95% CI)	p
CAD mRNA	M4	478	0.173 (0.092 to 0.255)	3.4×10^{-5}
CAD mRNA	M4 + ERG mRNA	478	0.078 (−0.005 to 0.161)	0.067
mTORC1 score	M4	478	0.117 (0.075 to 0.160)	1.1×10^{-7}
mTORC1 score	M4 + ERG mRNA	478	0.152 (0.108 to 0.197)	6.0×10^{-11}
Pyrimidine score	M4	478	0.074 (0.016 to 0.133)	0.013
Pyrimidine score	M4 + ERG mRNA	478	0.087 (0.025 to 0.150)	0.0065

(d) Cross-cohort ERG adjustment, per cohort.

Cohort	n	r(PTEN, ERG)	ERG-high fraction	Unadjusted (SE)	Adj. ERG mRNA (SE)	Adj. ERG-high (SE)
TCGA-PRAD	497	−0.27	0.40	0.178 (0.090)	0.060 (0.089)	0.057 (0.090)

Cohort	n	r(PTEN, ERG)	ERG-high fraction	Unadjusted (SE)	Adj. ERG mRNA (SE)	Adj. ERG-high (SE)
GSE21034	131	−0.14	0.56	−0.061 (0.174)	0.128 (0.160)	0.128 (0.162)
GSE6919	65	−0.28	0.31	0.130 (0.245)	−0.014 (0.243)	−0.001 (0.256)
GSE35988	49	−0.19	0.39	0.791 (0.281)	0.792 (0.283)	0.800 (0.280)
GSE46602	36	−0.40	0.58	0.375 (0.326)	0.297 (0.339)	0.290 (0.341)
GSE70770	219	−0.14	0.63	0.357 (0.135)	0.144 (0.107)	0.184 (0.091)
GSE55945	13	−0.13	0.54	−0.315 (0.518)	−0.346 (0.538)	−0.318 (0.544)
GSE32571	59	0.05	0.03	0.330 (0.257)	0.324 (0.258)	0.331 (0.259)

(e) Pooled (DL τ^2).

Analysis	Estimate	95% CI, Wald	p, Wald	95% CI, Hartung–Knapp	p, Hartung–Knapp	I^2 (%)
Unadjusted (model-based; = Table 4)	0.231	0.071 to 0.391	0.0046	0.032 to 0.430	0.029	27.2
Adjusted for ERG mRNA	0.144	0.015 to 0.273	0.029	−0.016 to 0.304	0.071	12.7
Adjusted for ERG-high cluster	0.162	0.032 to 0.292	0.015	0.001 to 0.323	0.049	16.2

All analyses in this table are post hoc. The decomposition and within-stratum models were run after the result of model M6 was known, without a prior written plan; the ERG-proxy validation, M4 + ERG mRNA and the ERG-adjusted cross-cohort analyses were specified in a plan addendum committed before they were run.

Table S11. Non-TCGA genomic cohort: MSKCC (Taylor et al.; same patients as GSE21034), primary tumors with copy number and expression (13 PTEN-deleted, of which 6 deep; 96 CN-neutral; models use the 108 tumors with a Gleason score).

Outcome	Mean, deleted	Mean, CN-neutral	Hedges' g (95% CI)	Wilcoxon p
CAD mRNA	8.019	7.939	0.347 (−0.23 to 0.93)	0.28
mTORC1 score	0.079	−0.000	0.221 (−0.36 to 0.80)	0.44
Pyrimidine score	0.068	−0.025	0.267 (−0.31 to 0.85)	0.38

Outcome	Model	n	β (95% CI)	p
CAD mRNA	Unadjusted	108	0.037 (−0.099 to 0.172)	0.59
CAD mRNA	Gleason-adjusted	108	0.027 (−0.110 to 0.164)	0.69
mTORC1 score	Unadjusted	108	0.062 (−0.156 to 0.280)	0.57
mTORC1 score	Gleason-adjusted	108	0.057 (−0.165 to 0.279)	0.61
Pyrimidine score	Unadjusted	108	0.071 (−0.141 to 0.282)	0.51
Pyrimidine score	Gleason-adjusted	108	0.051 (−0.164 to 0.265)	0.64

Signatures scored with GSVA within the cohort (mTORC1: 188 of 199 genes present; pyrimidine: 9 of 10). Same patients as GSE21034.

Table S12. CAD tumor-versus-normal differential expression by cohort (limma; all tumor samples versus normal; cell lines and xenografts excluded).

Cohort	n tumor/normal	CAD $\log_2$FC	SE	95% CI	p
TCGA-PRAD	498/52	0.325	0.065	0.198 to 0.452	7.7×10^{-7}
GSE21034	150/29	−0.845	0.140	−1.120 to −0.570	9.4×10^{-9}
GSE6919	90/81	0.117	0.044	0.030 to 0.204	0.0094
GSE35988	76/12	0.481	0.184	0.122 to 0.841	0.010
GSE46602	36/14	0.057	0.070	−0.080 to 0.195	0.42
GSE70770	219/74	−0.086	0.041	−0.166 to −0.007	0.034
GSE55945	13/8	0.272	0.194	−0.109 to 0.652	0.18
GSE32571	59/39	0.163	0.046	0.074 to 0.253	5.1×10^{-4}
Pooled, REML τ^2, Wald	—	0.057	—	−0.208 to 0.323	0.67
Pooled, REML τ^2, Hartung–Knapp	—	0.057	—	−0.272 to 0.386	0.69

I^2 = 97.0%, τ^2 = 0.135, Q_7 = 84.2, p = 1.9×10^{-15}. The TCGA-PRAD value here (HiSeqV2, limma) differs from the genome-wide limma-voom estimate in Figure 1A ($\log_2$FC 0.23) because the input matrix and model differ.

Table S13. Secondary, composition-mixed analysis: CAD mRNA in PTEN-Low versus PTEN-High samples using all samples in each cohort.

Cohort	n	n Low/High	Hedges' g (95% CI)	SE	p
TCGA-PRAD	550	275/275	0.301 (0.133 to 0.470)	0.086	4.3×10^{-4}
GSE21034	185	93/92	0.340 (0.049 to 0.630)	0.148	0.022
GSE6919	171	86/85	−0.217 (−0.518 to 0.083)	0.153	0.16
GSE35988	88	44/44	1.232 (0.776 to 1.689)	0.233	1.2×10^{-7}
GSE46602	50	25/25	0.129 (−0.426 to 0.684)	0.283	0.65
GSE70770	293	147/146	0.289 (0.059 to 0.519)	0.117	0.014
GSE55945	21	11/10	−0.247 (−1.107 to 0.613)	0.439	0.56
GSE32571	98	49/49	0.175 (−0.221 to 0.572)	0.202	0.38

Pooled estimates:

τ^2 estimator	Inference	g	95% CI	p	τ^2	I^2 (%)
FE	Wald z	0.267	0.164 to 0.371	4.3×10^{-7}	0.000	76.2
DL	Wald z	0.272	0.032 to 0.512	0.026	0.081	76.2
DL	Hartung–Knapp (t_7)	0.272	−0.080 to 0.624	0.11	0.081	76.2
REML	Wald z	0.271	−0.019 to 0.561	0.067	0.133	84.0
REML	Hartung–Knapp (t_7)	0.271	−0.091 to 0.632	0.12	0.133	84.0
PM	Wald z	0.270	−0.030 to 0.571	0.078	0.146	85.2
PM	Hartung–Knapp (t_7)	0.270	−0.093 to 0.633	0.12	0.146	85.2

$Q_7 = 29.38$, $p = 1.2\times10^{-4}$.

Table S14. GSE21034 sample composition by PTEN stratum (single median split across all 185 samples).

Sample category	PTEN-Low	PTEN-High	Mean CAD, Low	Mean CAD, High
Primary tumors	67	64	8.925	8.954
Normal prostate	20	9	9.680	9.520
Metastases	6	13	7.875	7.658
Cell lines/xenografts	0	6	—	7.866
Total	93	92		

Normal prostate falls predominantly into PTEN-Low and metastases and model-system samples into PTEN-High, which produces the positive all-sample effect (g = 0.34) that is absent in primary tumors (g = −0.06). The 66/65 split in Table 4 arises because the principal analysis recomputes the median within primary tumors.

Table S15. Robustness of the principal cross-cohort analysis.

(a) Leave-one-out (DerSimonian–Laird τ^2).

Cohort omitted	g	95% CI, Wald	p, Wald	95% CI, Hartung–Knapp	p, Hartung–Knapp	I^2 (%)
TCGA-PRAD	0.254	0.040 to 0.469	0.020	−0.013 to 0.521	0.059	30.0
GSE21034	0.258	0.127 to 0.388	1.1×10^{-4}	0.095 to 0.420	0.0083	0.8
GSE6919	0.241	0.063 to 0.418	0.0078	0.010 to 0.472	0.044	31.9
GSE35988	0.191	0.068 to 0.315	0.0023	0.050 to 0.332	0.016	0.0
GSE46602	0.221	0.050 to 0.392	0.011	−0.003 to 0.445	0.052	31.1
GSE70770	0.193	0.013 to 0.373	0.035	−0.039 to 0.425	0.088	21.2
GSE55945	0.238	0.081 to 0.395	0.0030	0.036 to 0.440	0.028	25.2
GSE32571	0.221	0.045 to 0.396	0.014	−0.009 to 0.450	0.057	31.4

(b) Subgroups. Meta-regression on log cohort size: slope −0.030 (SE 0.092), p = 0.74. Egger's test p = 0.83 (k = 8; uninformative).

Subset	k	g	95% CI, Wald	p, Wald	p, Hartung–Knapp	I^2 (%)
Microarray only	7	0.254	0.040 to 0.469	0.020	0.059	30.0
n>=100 primary	3	0.179	−0.016 to 0.374	0.071	0.23	44.1
n<100 primary	5	0.336	0.054 to 0.618	0.020	0.081	9.0

(c) PTEN–CAD Pearson correlations, pooled on the Fisher z scale.

Analysis	Cohort	n	r	p
All samples	TCGA-PRAD	550	−0.262	4.7×10^{-10}
All samples	GSE21034	185	−0.254	4.8×10^{-4}
All samples	GSE6919	171	0.068	0.38
All samples	GSE35988	88	−0.577	4.0×10^{-9}
All samples	GSE46602	50	−0.077	0.60
All samples	GSE70770	293	−0.093	0.11
All samples	GSE55945	21	0.283	0.21
All samples	GSE32571	98	−0.122	0.23
Primary tumours	TCGA-PRAD	497	−0.217	1.0×10^{-6}
Primary tumours	GSE21034	131	−0.054	0.54
Primary tumours	GSE6919	65	−0.134	0.29
Primary tumours	GSE35988	49	−0.508	1.9×10^{-4}
Primary tumours	GSE46602	36	−0.053	0.76
Primary tumours	GSE70770	219	−0.131	0.053
Primary tumours	GSE55945	13	0.189	0.54
Primary tumours	GSE32571	59	−0.062	0.64
All samples	Pooled (DL τ^2)	—	−0.168	Wald 0.020 [−0.302 to −0.027]; HK 0.096 [−0.361 to 0.039]; I^2 83.3%
Primary tumours	Pooled (DL τ^2)	—	−0.158	Wald 0.0015 [−0.252 to −0.061]; HK 0.023 [−0.282 to −0.029]; I^2 45.3%

Table S16. ERG measurement by cohort (primary tumors) and ERG-adjusted cross-cohort analysis without GSE32571 (post hoc).

Cohort	ERG features	ERG IQR (log_2)	r(ERG, CACNA1D)	p	r(ERG, target composite)	ERG-high (%)
TCGA-PRAD	RSEM gene-level ERG	4.83	0.68	1.9×10^{-67}	0.86 (5 genes)	40
GSE21034	Exon array, gene-level ERG	0.58	0.87	1.5×10^{-41}	0.07 (3 genes)	56
GSE6919	HG-U95Av2 probesets annotated ERG (e.g. 2046_at, 36383_at, 913_at, 914_g_at)	1.24	0.42	4.5×10^{-4}	0.66 (3 genes)	31
GSE35988	Agilent A_23_P301414, A_23_P57323, A_24_P922378	3.20	0.69	5.1×10^{-8}	0.81 (5 genes)	39
GSE46602	HG-U133 Plus 2: 211626_x_at, 213541_s_at, 222079_at, 241926_s_at	5.35	0.71	1.0×10^{-6}	0.86 (5 genes)	58
GSE70770	HumanHT-12 v4: ILMN_1727426, ILMN_1768301, ILMN_1785486	0.41	0.77	1.6×10^{-43}	0.75 (5 genes)	63
GSE55945	HG-U133 Plus 2: 211626_x_at, 213541_s_at, 222079_at, 241926_s_at	2.85	0.65	0.016	0.70 (5 genes)	54
GSE32571	HumanHT-12 v3: ILMN_1727426, ILMN_1768301, ILMN_1785486	0.12	−0.01	0.97	0.11 (5 genes)	3

Pooled estimates without GSE32571 (k = 7; DL τ^2):

Analysis	Estimate	Wald 95% CI	Wald p	Hartung–Knapp 95% CI	Hartung–Knapp p	I^2 (%)
Unadjusted (model-based)	0.224	0.043 to 0.405	0.015	−0.016 to 0.464	0.062	36.3
Adjusted for ERG mRNA	0.136	−0.006 to 0.278	0.061	−0.051 to 0.323	0.12	19.4
Adjusted for ERG-high cluster	0.154	0.011 to 0.298	0.035	−0.035 to 0.343	0.093	23.6

Target composite: mean Z-score of the available ERG-fusion target genes among TDRD1, PLA1A, CACNA1D, KCNS3 and CRISP3. Where several features map to ERG, the cohort's symbol-level value was used. In GSE32571 all three Illumina ERG probes (ILMN_1727426, ILMN_1768301, ILMN_1785486) had median values of 5.9–6.3 log_2 units in the series-matrix data, close to the array background, with interquartile ranges of 0.13–0.31, and none correlated consistently with the target genes (probe-level r from −0.22 to 0.43), so ERG adjustment is uninformative in that cohort. In GSE21034 ERG correlated strongly with CACNA1D but negatively with CRISP3, which lowers its composite correlation.

Table S17. Progression-free interval (TCGA Pan-Cancer Clinical Data Resource PFI; Cox models; pre-specified, exploratory; median follow-up 30.9 months, 95% CI 28.6–34.9, reverse Kaplan–Meier).

Exposure	Adjustment	n	Events	HR (95% CI)	p
PTEN deletion	none	482	90	1.56 (1.03–2.36)	0.037
PTEN deletion	grade group, pT	475	89	1.02 (0.66–1.57)	0.92
CAD mRNA (per SD)	none	482	90	1.44 (1.16–1.78)	9.2×10^{-4}
CAD mRNA (per SD)	grade group, pT	475	89	1.18 (0.95–1.46)	0.13
Pyrimidine score (per SD)	none	482	90	1.49 (1.20–1.84)	2.7×10^{-4}
Pyrimidine score (per SD)	grade group, pT	475	89	1.16 (0.93–1.44)	0.18
mTORC1 score (per SD)	none	482	90	0.95 (0.77–1.17)	0.63
mTORC1 score (per SD)	grade group, pT	475	89	0.93 (0.76–1.13)	0.47

Table S18. GSE138417 (MCF7): MLN0128 versus DMSO by RNA fraction.

Gene	Cytosolic $\log_2$FC	FDR	Polysome $\log_2$FC	FDR	Polysome − cytosolic	FDR
CAD	−0.19	0.68	0.26	0.46	0.46	0.74
DHODH	−0.64	0.077	−0.70	0.033	−0.05	1.00
UMPS	−0.77	0.024	−1.14	2.9×10^{-4}	−0.37	0.82
CTPS1	−0.95	0.0051	−1.12	4.3×10^{-4}	−0.17	0.97
CTPS2	0.33	0.52	−0.59	0.13	−0.92	0.53
RRM1	0.12	0.86	−0.26	0.56	−0.38	0.88
RRM2	−0.97	0.0061	−1.61	3.5×10^{-6}	−0.63	0.63
TK1	−0.81	0.0088	−0.85	0.0032	−0.04	1.00
TYMS	−0.65	0.068	−0.82	0.010	−0.17	0.97
DTYMK	−0.86	0.0037	−0.69	0.013	0.17	0.96
NME1	−0.55	0.065	−0.47	0.082	0.08	0.99
RPS6	0.15	0.72	−1.63	9.2×10^{-8}	−1.78	1.3×10^{-4}
RPS6KB1	−0.01	0.99	−0.82	0.042	−0.80	0.61
EIF4E	−0.45	0.36	−1.04	0.0071	−0.59	0.71
EIF4EBP1	−0.77	0.029	−0.71	0.027	0.05	1.00
MTOR	−0.09	0.88	0.03	0.96	0.12	0.99
RPTOR	−0.49	0.30	−0.59	0.14	−0.10	0.99
MLST8	−0.39	0.29	−0.37	0.24	0.02	1.00

limma with fraction × treatment term and replicate block; 13,982 genes; genome-wide FDR within each contrast. Camera p for the pyrimidine set: cytosolic 4.3×10^{-4}, polysome 1.7×10^{-3}, difference 0.98.

Table S19. Characteristics of the analyzed TCGA-PRAD subsets compared with all classified primary tumors (post hoc, descriptive).

Subset	n	PTEN-deleted (%)	Deep deletion (%)	Mean age	Grade group 4–5 (%)	pT3–T4 (%)	Mean CAD ($\log_2$)	p, PTEN status	p, grade group	p, pT	p, CAD
All classified primary tumours	485	32.8	19.6	61.0	41.0	61.3	9.74				
RPPA subset	342	34.5	21.1	61.5	42.1	68.0	9.76	0.24	0.16	3.3×10^{-5}	0.17
Classified tumours without RPPA	143	28.7	16.1	59.8	38.5	45.4	9.70				
TCGA 2015 study tumours	327	33.6	19.6	60.7	38.5	60.6	9.83	0.61	0.044	0.24	1.2×10^{-9}
M6 complete-case subset	276	37.7	21.7	60.5	38.8	59.8	9.84	0.0085	0.29	0.31	2.2×10^{-8}

Subset	n	PTEN-deleted (%)	Deep deletion (%)	Mean age	Grade group 4–5 (%)	pT3–T4 (%)	Mean CAD (log_2)	p, PTEN status	p, grade group	p, pT	p, CAD
Classified tumours outside M6 subset	209	26.3	16.7	61.6	44.0	63.4	9.61				

p-values compare the subset with the classified tumors outside it (Fisher's exact test for PTEN status; Wilcoxon test otherwise). The TCGA 2015 study tumors had higher CAD mRNA than the remainder, which may reflect sequencing batch.

Supplementary Note S1. Exploratory Cell-Line Western Blots (Single Experiments; Not Used for Any Conclusion of the Main Manuscript)

These experiments were reported in the main text of an earlier version of this manuscript. In line with the reviewers' requests, they are now presented here as exploratory observations. Each panel has been rebuilt directly from the original image files (Figure S7), and no densitometry is reported.

N1.1. Cell Lines

Five human prostate epithelial lines of common parental origin were used: the HPV-18-immortalized parental line RWPE-1 (ATCC CRL-11609, now supplied as CRL-3607; RRID:CVCL_3791) [46] and four derivatives generated from it by N-methyl-N-nitrosourea exposure and selection: WPE1-NA22 (CRL-2849; RRID:CVCL_3810), WPE1-NB14 (CRL-2850; RRID:CVCL_3812), WPE1-NB11 (CRL-2851; RRID:CVCL_3811) and WPE1-NB26 (CRL-2852; RRID:CVCL_3813) [47]. The depositor ranks the derivatives by increasing malignancy as NA22 < NB14 < NB11 < NB26 [47]. The lines share a parental origin but differ at an uncharacterized number of loci. They are not isogenic for any single gene. No alteration of the PTEN locus (10q23) is reported in their genomic characterization [47,48], and PTEN protein is retained in the parental line [49]. **This series is therefore not a PTEN-loss model and was not used as one.** PTEN was not measured in these experiments, and the ERG/ETS-fusion status of the lines is not documented. The lines were obtained from the ATCC and used within ten passages of receipt; the exact passage number was not recorded, and no in-house short-tandem-repeat authentication was performed. Cells were grown in Keratinocyte Serum-Free Medium with bovine pituitary extract (0.05 mg/mL) and EGF (5 ng/mL) at 37 °C in 5% CO_2.

N1.2. Western Blotting

Cells were lysed in RIPA buffer. Equal amounts of protein were resolved by SDS-PAGE, transferred to PVDF membranes, probed with primary antibodies against pCAD (Ser1859), total CAD, phospho-S6K1 (Thr389), total S6K1 and β-actin, then with HRP-conjugated secondary antibodies, and detected by enhanced chemiluminescence. Antibody catalogue and lot numbers, dilutions and exposure times were not recorded in the files available to us.

Membranes. As shown by the original image files (Figure S7):

- pCAD (Ser1859) and total CAD for the baseline panel were detected on two separate membranes that were imaged together in one file (Figure S7A, upper and lower). The two antibodies detect the same protein (about 243 kDa), so the two signals cannot come from one membrane cut by molecular weight.
- Phospho-S6K1 and total S6K1 were detected on **separate membranes**, so no phospho/total ratio from the same membrane is available.
- β-actin for the baseline panel is on a further membrane.
- In the fisetin experiment, pCAD (Ser1859) and total CAD for each line were detected on separate membranes (Figure S7E, F).

- Each line is shown with **its own β-actin** (Figure S7G). β-actin was detected after reprobing the pCAD (Ser1859) membrane of the same line.

Image files. The available image files are exports without acquisition metadata; native imager files were not located. They are low-resolution exports (348 × 260 to 432 × 321 pixels), which limits the resolution of Figures S5–S7.

Unused lanes and markers. - Lanes to the right of the outlined regions in Figure S7A (pCAD and total CAD) and Figure S7D (β-actin) were not part of the experiments reported here, and their contents are not described in the laboratory records available to us. - Molecular-weight markers are visible on the baseline β-actin membrane (Figure S7D) and on the fisetin membranes (Figure S7E–G). No markers are visible on the baseline pCAD, total CAD, phospho-S6K1 and total S6K1 membranes (Figure S7A–C), so band identity on those membranes rests on the antibody used.

N1.3. Fisetin Treatment

Fisetin was used because it has been reported to suppress mTOR signaling in prostate cancer cells [50,51]. WPE1-NB26 and WPE1-NB11, the two most malignant derivatives in the depositor's ranking [47], were treated with fisetin (Sigma-Aldrich; in DMSO, final ≤ 0.1% v/v, matched in vehicle controls) at 0, 20, 40 and 60 μM for 48 h [50,51]. These concentrations are far above achievable plasma levels (peak ≈ 0.04–0.8 μM in humans [52]). Cell viability was not assessed.

N1.4. Observations

Baseline panel. In a single experiment, the pCAD (Ser1859) band pattern of RWPE-1 and WPE1-NA22 differed from that of the other three lines (Figure S5), as did the migration of total CAD. pCAD signal and CAD abundance therefore cannot be compared across the series from these membranes. Phospho-S6K1 (Thr389) and total S6K1 were detected in all five lines on separate membranes, and β-actin was broadly similar across lanes.

Fisetin. pCAD (Ser1859) signal varied with fisetin concentration in both lines (Figure S6). In WPE1-NB11, β-actin was very weak at 40 μM and weaker at 60 μM than at 0–20 μM, in the lanes with the weakest pCAD signal. In the same line, total CAD also varied: it was weak at 40 μM, and at 60 μM a strong, slower-migrating band appeared. In WPE1-NB26, β-actin also varied between lanes.

Interpretation. These single experiments:

- have no biological replicates, viability controls or upstream pathway readouts;
- cannot distinguish an effect on mTOR–S6K1 signaling from altered CAD abundance, an effect on cell health, or unequal loading;
- allow no statistical comparison.

They are reported for completeness and transparency and are not used for any conclusion of the main manuscript.

Corrections relative to the earlier version.

- Former Figure 9 showed a single β-actin strip, taken from the WPE1-NB26 membrane, for both cell lines. Figure S6 shows each line's own β-actin.
- The total CAD strip of former Figure 8 could not be matched to the original image file and has been replaced by the corresponding region of that file.
- The densitometric values reported earlier (former Table S7) could not be reproduced from the available image files and are withdrawn.

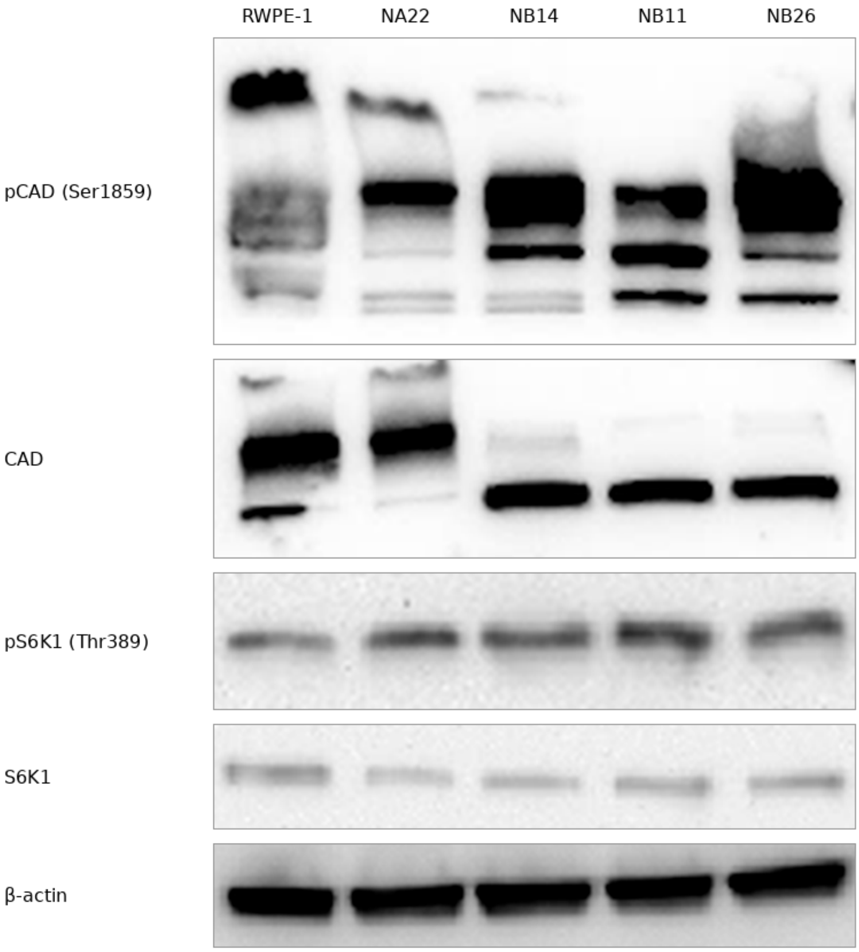

Figure S5. Exploratory baseline Western blot (single experiment; Supplementary Note S1). pCAD (Ser1859), total CAD, phospho-S6K1 (Thr389), total S6K1 and β-actin in the parental RWPE-1 line and four WPE1 derivatives (NA22, NB14, NB11, NB26). Each strip is a single continuous rectangular region of the original image file (outlined in Figure S7A–D), scaled uniformly, without splicing or brightness/contrast adjustment; grey levels are those of the original files, and strips are enlarged about 3–4-fold relative to Figure S7. Phospho- and total S6K1 are on separate membranes. PTEN was not measured; the series is not a PTEN-loss model.

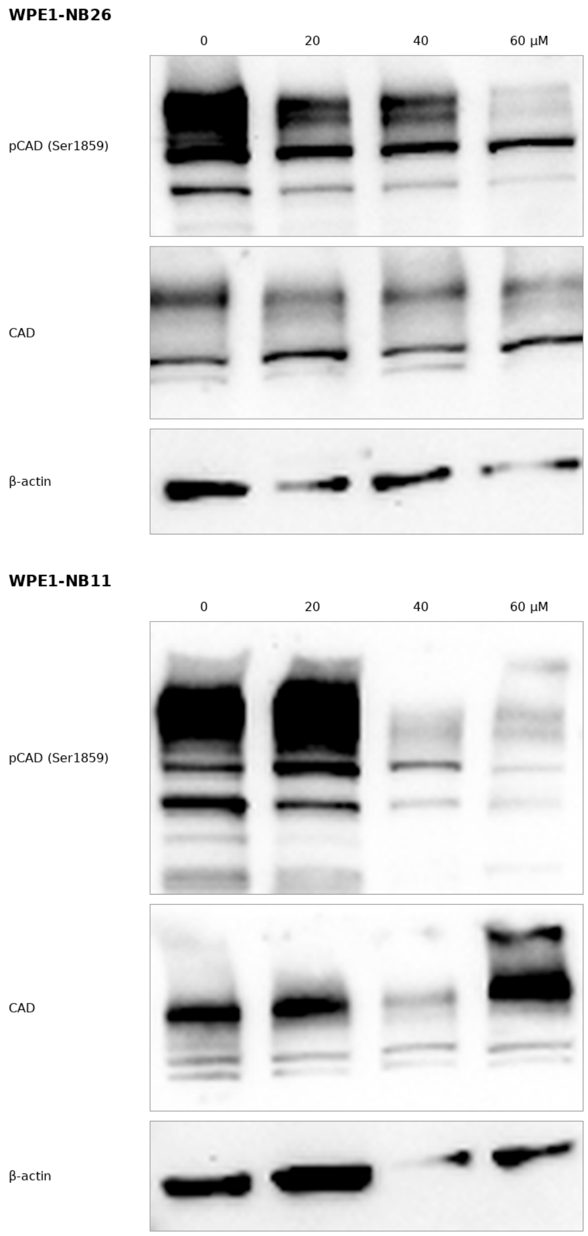

Figure S6. Exploratory fisetin treatment (single experiment per line; Supplementary Note S1). pCAD (Ser1859), total CAD and β-actin in WPE1-NB26 and WPE1-NB11 treated with fisetin (0, 20, 40, 60 µM, 48 h). Each line is shown with its own β-actin (Figure S7G). Strips are continuous regions of the original image files (outlined in Figure S7E–G), scaled uniformly, without splicing or brightness/contrast adjustment; grey levels are those of the original files, and strips are enlarged relative to Figure S7. β-actin and total CAD vary between lanes. In WPE1-NB11, β-actin is very weak at 40 µM and weaker at 60 µM than at 0–20 µM, in the lanes with the weakest pCAD signal. No densitometry or statistical comparison is reported. Cell viability was not assessed.

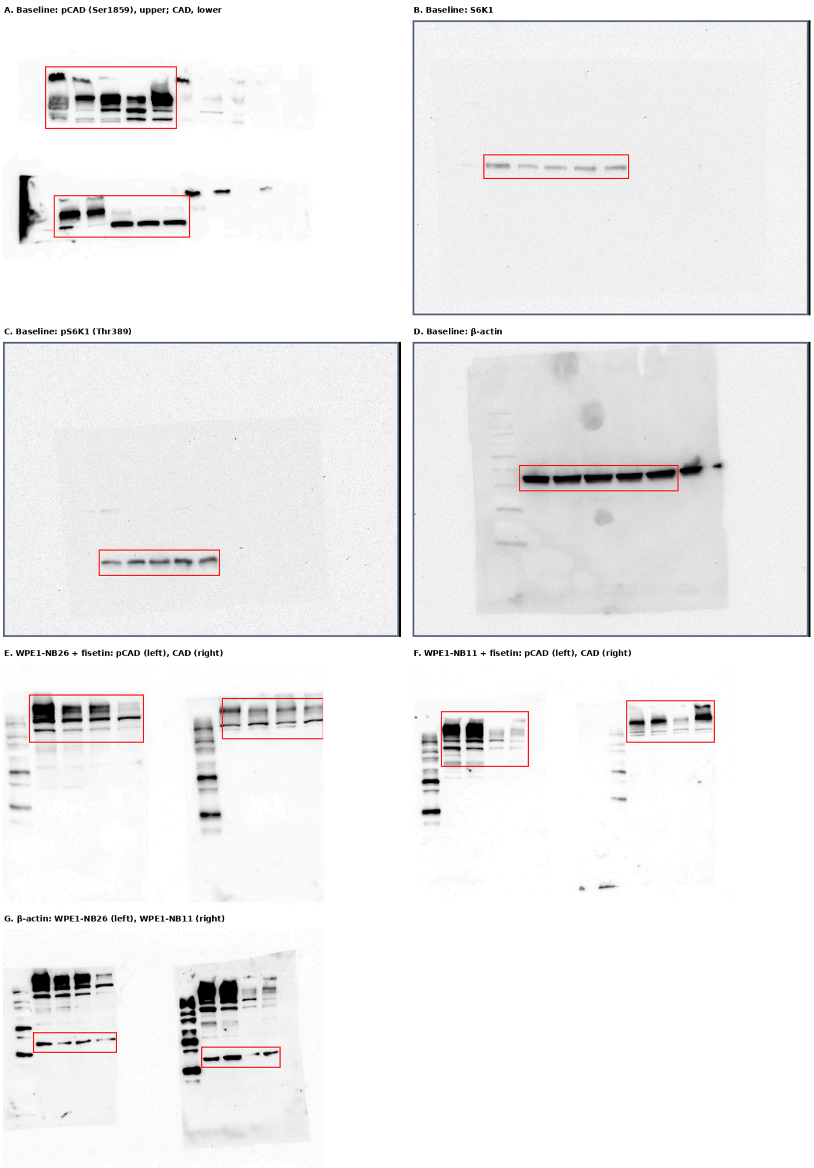

Figure S7. Original, uncropped Western blot image files for Figures S5 and S6. (A) pCAD (Ser1859), upper, and total CAD, lower: two separate membranes imaged together (baseline panel); (B) total S6K1; (C) phospho-S6K1 (Thr389); (D) β-actin, with molecular-weight ladder; (E) WPE1-NB26, pCAD (Ser1859) left and total CAD right; (F) WPE1-NB11, pCAD (Ser1859) left and total CAD right; (G) β-actin for WPE1-NB26 (left membrane) and WPE1-NB11 (right membrane). Red rectangles, added here, mark the regions shown in Figures S5 and S6; the image content is otherwise unaltered. Lanes outside the rectangles in (A) and (D) are not part of this study. Molecular-weight markers are visible only in (D)–(G). The files are low-resolution exports without acquisition metadata.

References for Supplementary Note S1

46. Bello D, Webber MM, Kleinman HK, Wartinger DD, Rhim JS. Androgen responsive adult human prostatic epithelial cell line immortalized by human papillomavirus 18. Carcinogenesis 1997, 18, 1215–1223. https://doi.org/10.1093/carcin/18.6.1215
47. Webber MM, Quader ST, Kleinman HK, Bello-DeOcampo D, Storto PD, Bice G, DeMendonca-Calaca W, Williams DE. Human cell lines as an in vitro/in vivo model for prostate carcinogenesis and progression. Prostate 2001, 47, 1–13. https://doi.org/10.1002/pros.1041
48. Malhotra A, Shibata Y, Hall IM, Dutta A. Chromosomal structural variations during progression of a prostate epithelial cell line to a malignant metastatic state inactivate the NF2, NIPSNAP1, UGT2B17, and LPIN2 genes. Cancer Biol Ther 2013, 14, 840–852. https://doi.org/10.4161/cbt.25329

49. Kimbrough-Allah MN, Millena AC, Khan SA. Differential role of PTEN in transforming growth factor β (TGF-β) effects on proliferation and migration in prostate cancer cells. Prostate 2018, 78, 377–389. https://doi.org/10.1002/pros.23482
50. Khan N, Afaq F, Syed DN, Mukhtar H. Fisetin, a novel dietary flavonoid, causes apoptosis and cell cycle arrest in human prostate cancer LNCaP cells. Carcinogenesis 2008, 29, 1049–1056. https://doi.org/10.1093/carcin/bgn078
51. Suh Y, Afaq F, Khan N, Johnson JJ, Khusro FH, Mukhtar H. Fisetin induces autophagic cell death through suppression of mTOR signaling pathway in prostate cancer cells. Carcinogenesis 2010, 31, 1424–1433. https://doi.org/10.1093/carcin/bgq115
52. Krishnakumar IM, Jaja-Chimedza A, Joseph A, Balakrishnan A, Maliakel B, Swick A, Ittiyavirah S. Enhanced bioavailability and pharmacokinetics of a novel hybrid-hydrogel formulation of fisetin orally administered in healthy individuals: a randomised double-blinded comparative crossover study. J Nutr Sci 2022, 11, e74. https://doi.org/10.1017/jns.2022.72
